# Author Correction: Identifying long-term stable refugia for relict plant species in East Asia

**DOI:** 10.1038/s41467-018-07727-4

**Published:** 2018-12-04

**Authors:** Cindy Q. Tang, Tetsuya Matsui, Haruka Ohashi, Yi-Fei Dong, Arata Momohara, Sonia Herrando-Moraira, Shenhua Qian, Yongchuan Yang, Masahiko Ohsawa, Hong Truong Luu, Paul J. Grote, Pavel V. Krestov, Ben LePage, Marinus Werger, Kevin Robertson, Carsten Hobohm, Chong-Yun Wang, Ming-Chun Peng, Xi Chen, Huan-Chong Wang, Wen-Hua Su, Rui Zhou, Shuaifeng Li, Long-Yuan He, Kai Yan, Ming-Yuan Zhu, Jun Hu, Ruo-Han Yang, Wang-Jun Li, Mizuki Tomita, Zhao-Lu Wu, Hai-Zhong Yan, Guang-Fei Zhang, Hai He, Si-Rong Yi, Hede Gong, Kun Song, Ding Song, Xiao-Shuang Li, Zhi-Ying Zhang, Peng-Bin Han, Li-Qin Shen, Diao-Shun Huang, Kang Luo, Jordi López-Pujol

**Affiliations:** 1 Institute of Ecology and Geobotany, Yunnan University, 650091 Kunming, China; 2 Forestry and Forest Products Research Institute, Forest Research and Management Organization, Matsunosato 1, Tsukuba-shi, 305-8687 Ibaraki-ken, Japan; 3 Graduate School of Horticulture, Chiba University, 648 Matsudo, 271-8510 Chiba, Japan; 4 Botanic Institute of Barcelona (IBB, CSIC-ICUB), Passeig del Migdia s/n, 08038 Barcelona, Catalonia Spain; 5 Key Laboratory of Three Gorges Reservoir Region’s Eco-Environment, Ministry of Education, Chongqing University, 400045 Chongqing, China; 6 The Nature Conservancy Society of Japan, Mitoyo Bldg. 2F, 1-16-10 Shinkawa, Chuo-ku, 104-0033 Tokyo, Japan; 7 Southern Institute of Ecology, Vietnam Academy of Science and Technology, Ho Chi Minh City, Vietnam; 8 Northeastern Research Institute of Petrified Wood and Mineral Resources, Nakhon Ratchasima Rajabhat University, 30000 Nakhon Ratchasima, Thailand; 9 Botanical Garden-Institute FEB RAS, Makovskii Str. 142, 690024 Vladivostok, Russia; 10 Pacific Gas and Electric Company, 3401 Crow Canyon Road, 94583 San Ramon, CA USA; 11 The Academy of Natural Science, 1900 Benjamin Franklin Parkway, 19103 Philadelphia, PA USA; 12 Plant Ecology & Biodiversity, Utrecht University, Domplein 29, 3512 JE Utrecht, Netherlands; 13 Tall Timbers Research Station and Land Conservancy, 13093 Henry Beadel Drive, 32312 Tallahassee, FL USA; 14 Interdisciplinary Institute of environmental, Social and Human Studies, University of Flensburg, Flensburg, Germany; 15 Institute of Botany, Yunnan University, 650091 Kunming, Yunnan China; 16 Research Institute of Resource Insects, Chinese Academy of Forestry, 650224 Kunming, China; 17 Kunming Institute of Forestry Exploration and Design, The State Forestry Administration of China, 650216 Kunming, China; 18 Centre for Mountain Ecosystem Studies, Kunming Institute of Botany-CAS, 650204 Kunming, China; 19 CAS Key Laboratory of Mountain Ecological Restoration and Bioresource Utilization & Ecological Restoration and Biodiversity Conservation Key Laboratory of Sichuan Province, Chengdu Institute of Biology, Chinese Academy of Sciences, 610041 Chengdu, China; 20 Kunming Agrometeorological Station of Yunnan Province, 650228 Kunming, China; 21 Guizhou University of Engineering Science, 551700 Bijie, China; 22 Tokyo University of Information Sciences, 4-1 Onaridai Wakaba-ku, 265-8501 Chiba, Japan; 23 College of Life Sciences, Chongqing Normal University, Shapingba, 401331 Chongqing, China; 24 Chongqing Three Gorges Medical College, 404120 Chongqing, China; 25 School of Geography, Southwest China Forestry University, 650224 Kunming, China; 26 School of Ecological and Environmental Sciences, East China Normal University, 200241 Shanghai, China; 27 Kunming University of Science and Technology, 650500 Chenggong, China; 28 Yunnan Academy of Forestry, 650201 Kunming, China; 29 Key Laboratory of Tropical Forest Ecology, Xishuangbanna Tropical Botanical Garden, Chinese Academy of Sciences, Ailaoshan Station for Subtropical Forest Ecosystem Studies, National Forest Ecosystem Research Station at Ailaoshan, 650091 Kunming, Yunnan China

Correction to: *Nature Communications* 10.1038/s41467-018-06837-3; published online 26 October 2018.

In the original version of this Article, an incorrect sample size was provided for the number of relict species (443 instead of 442) and the number of relict forests (423 instead of 422). These errors have been corrected in both the PDF and HTML versions of the Article.

