# Supplementary material for: Identifying long-term stable refugia for relict plant species in East Asia
Source: Nat Commun. 2018 Oct 26;9:4488. doi: 10.1038/s41467-018-06837-3 (PMC6203703; doi:10.1038/s41467-018-06837-3)
Supplement: Supplementary file 1 — Supplementary Information [file 41467_2018_6837_MOESM1_ESM.pdf]

Supplementary Information

Tang et al. 2018. Identifying long-term stable refugia for relict plant species in East Asia.

Supplementary Note 1 Relict genera: their distribution in space and time before the LGM

This section presents mega/macro fossil records of the genera involved in space and time to provide a phytogeographic history of these genera.

The relict genera, both the endemic and those having disjunct distributions of fossil records, evidently had broad distribution ranges during the Paleogene and the Neogene Periods, and some of them even in the Cretaceous (Supplementary Figs. 5A and 5B). The relevant Triassic and Jurassic fossils, as represented by *Ginkgo*, were widely distributed around the world (Laurasia and Gondwana), with more occurrences being found in Eurasia (Supplementary Figs. 5A and 5B). East Asia, Europe and North America had much stronger floristic interrelationships in the Paleogene-Neogene and the Cretaceous than in later times. Among the 63 genera confirmed by fossil records endemic today to East Asia (Supplementary Table 1), 55 exhibit fossils in Asia, 49 in Europe, and 33 in North America. Among the 36 genera confirmed by fossil records with disjunct distributions (Supplementary Table 2), 34 have fossil records in Asia, 33 in Europe, and 31 in North America. Of the genera whose origins can be found in the Arcto-Tertiary or boreotropical flora, many dominated the Northern Hemisphere during the Paleogene and the Neogene. Most of the relict genera that are presently endemic to East Asia also occurred in North America and/or Europe before the Pleistocene (Supplementary Table 1)[also 1]. The present endemism was formed when some dispersal routes became discontinuous, such as the North Atlantic land bridge and areas along the Tethys Seaway in the Paleogene and the much colder and drier conditions precluding the use of the Bering land bridge by temperate floral elements by late Neogene time and for all plants due to glaciation in the Quaternary[2,3].

Among the 63 endemic genera, a higher number of genera of deciduous broad-leaved woody plants first appeared in the Paleocene and especially in the Miocene in Asia, whereas in both Europe and North America most of the genera of this type of woody plants first appeared in the Eocene (Supplementary Figs. 6A–6C). Of the disjunct genera a higher number first appeared in the Eocene in Asia (especially deciduous broad-leaved taxa) and North America, while the disjunct genera mostly first appeared in the Oligo-Miocene in Europe (Supplementary Figs. 6D–6F). This is consistent with the geological history of Asia, Europe and North America. The mountain

ranges (e.g. the Himalayas in Asia, the Alps in Europe, the Rocky Mountains in North America) were all being uplifted starting from about the Paleocene/Eocene in Asia, about the Eocene in Europe, and about the Paleocene in North America. Most of them had become high mountains by/in the Miocene. The formation of the Asian monsoons has been attributed to the uplift of the Himalayas and Tibetan plateau[4,5] as well as changes in the land-sea distribution[6]. The uplift has obstructed humid air currents from going deep into northern China. Southern, southwestern and southeastern China have enhanced moisture from sea breezes. Thus subtropical China has been suitable for moisture-requiring relict plant species.

Many species of the relict genera became extinct in Europe and North America as tropical/sub-tropical environments there became temperate. The extinction of endemic taxa peaked in the Eocene (e.g. *Cryptomeria*, *Corylopsis*, *Diplopanax, Toricellia*) and Miocene (e.g. *Cathaya*, *Cephalotaxus*, *Cunninghamia*, *Eucommia*, *Ginkgo*, *Glyptostrobus*, *Pseudolarix*) in North America, possibly caused by terminal Eocene and late Miocene climatic deterioration, while extinction occurred mainly in the Mio-Pliocene (e.g. including the taxa just mentioned above, and additionally e.g. *Nothotsuga*, *Phellodendron*, *Taiwania*) in Europe (Supplementary Figs. 6G–6J), where the oceanic climate with humid conditions continued until the middle Pliocene[7,8].

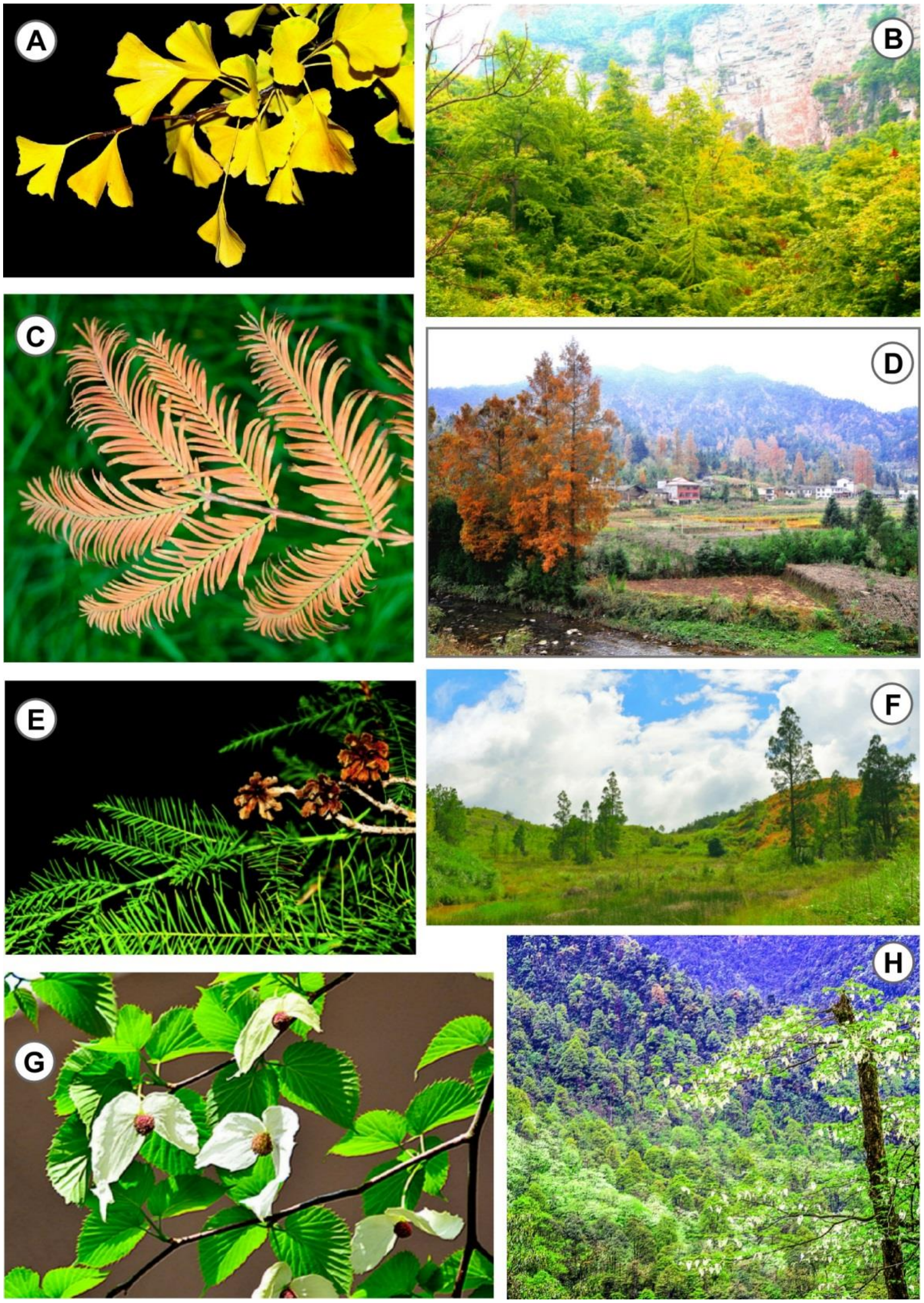

Supplementary Fig. 1 Symbolic relict species of East Asia. (A) Foliage of *Ginkgo biloba*. (B) A wild *G. biloba* forest in Yanjiagou, Nanchuan of Chongqing Municipality, southwestern China. (C) Foliage of *Metasequoia glyptostroboides*. (D) A wild *M. glyptostroboides* forest in the Shuisha Valley, Zhonglu, Lichuan, south-central China. (E) Foliage and seed cones of *Glyptostrobus pensilis*. (F) A wild *G. pensilis* swamp community in Nanping, Fujian, southeastern China. (G) Inflorescences of *Davidia involucrata*. (H) A *D. involucrata* forest in Longcanggou, Sichuan, southwestern China.
Photographs: (A), (B), (C), (E), (F) & (G) by Cindy Q. Tang; (D) by Yongchuan Yang; (H) by Shi-Liu Wang

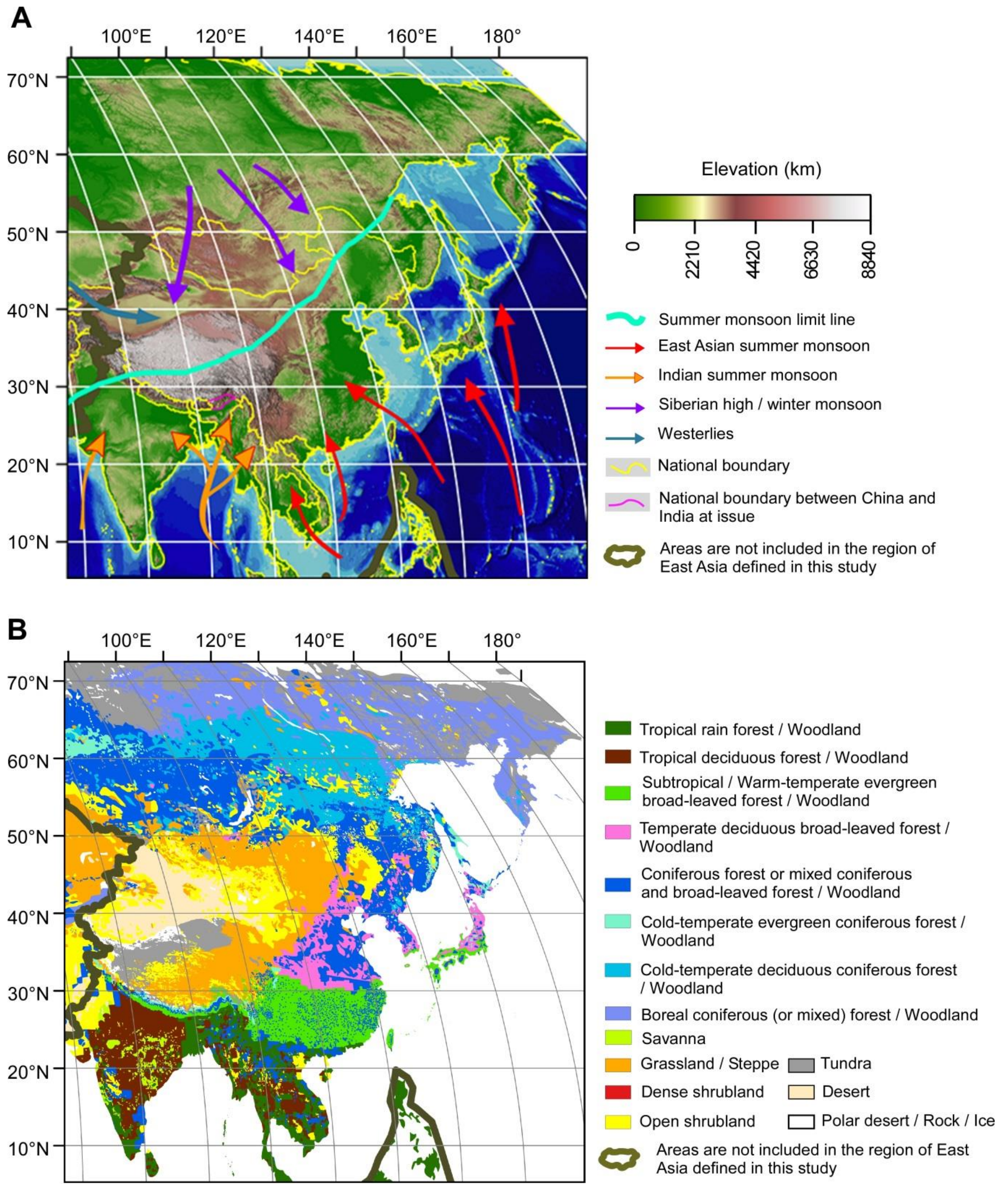

Supplementary Fig. 2 Land, climate and vegetation of East Asia. (A) Topography and climate system. The summer monsoon limit line adapted from Chen et al.[9], showing the approximate extent of modern summer monsoon precipitation. (B) Potential vegetation types of East Asia (agricultural land, buildings, etc. are not taken into account) revised from Ramankutty and Foley[10]. Woodland represents undefined woody-mixed communities. Maps were generated using the software ArcGIS v. 10.5 (ESRI, Redlands, CA, USA) and modified using Canvas 12 (ACD Systems of America, Inc., Seattle, WA, USA). Map layers were obtained from site www.gadm.org.

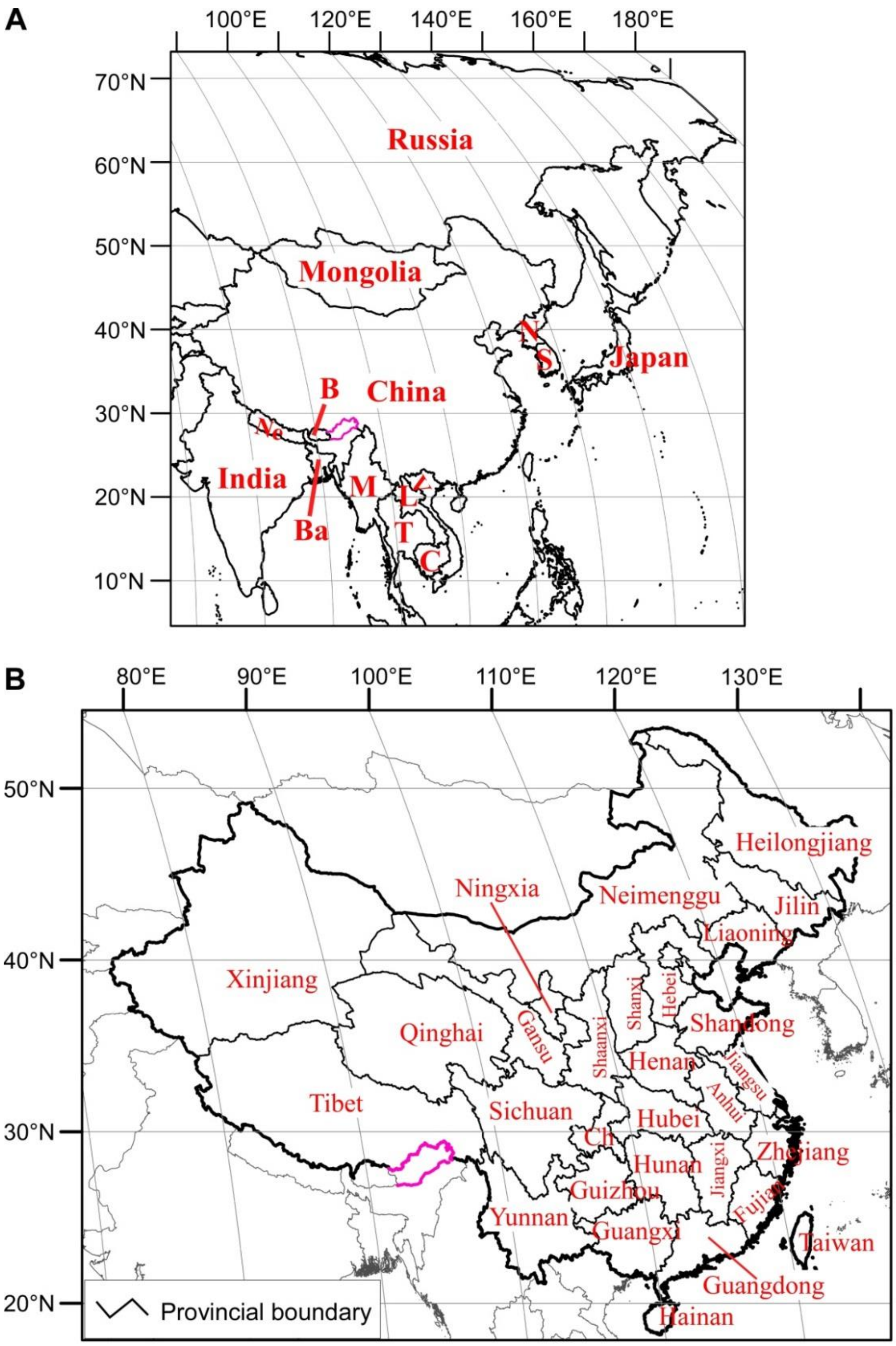

Supplementary Fig. 3 East Asia and provinces of China. (A) Countries of East Asia defined in this study. B=Bhutan; Ne=Nepal; Ba=Bangladesh; M=Myanmar; L=Laos; T=Thailand; C=Cambodia; V=Vietnam; N=North Korea; S=South Korea. (B) Provinces of China. Ch = Chongqing. Purple lines: national boundaries between China and India at issue. Maps were generated using the software ArcGIS v. 10.5 (ESRI, Redlands, CA, USA) and modified using Canvas 12 (ACD Systems of America, Inc., Seattle, WA, USA). Map layers were obtained from site www.gadm.org.

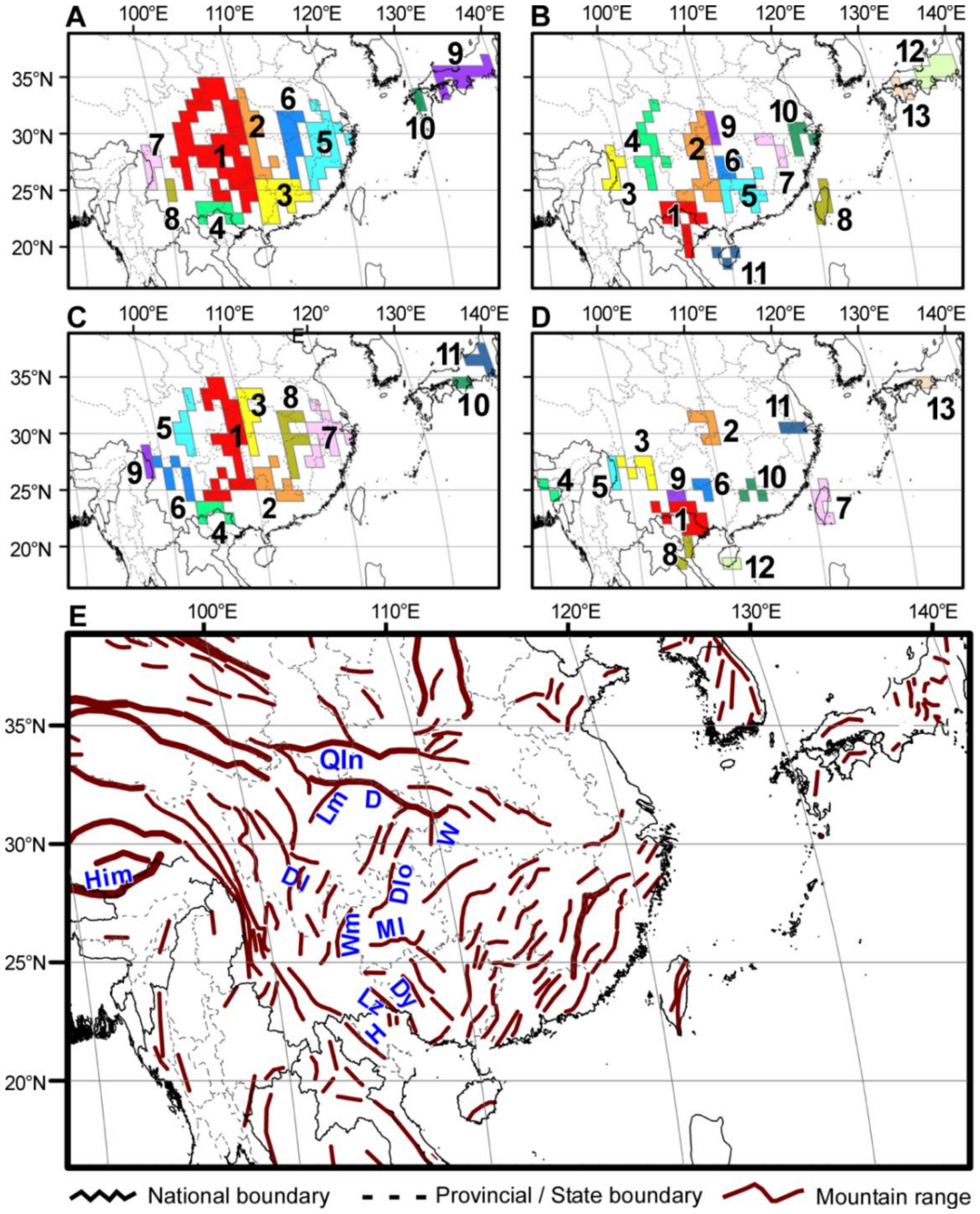

Supplementary Fig. 4 Core areas of relict species richness and rarity-weighted richness (RWR) in East Asian mountain systems. (A) Core areas of relict species richness of endemic genera. (B) Core areas of relict species rarity-weighted richness of endemic genera. (C) Core areas of relict species richness of disjunct genera. (D) Core areas of relict species rarity-weighted richness of disjunct genera. (E) Major mountain ranges of East Asia. Only the names of mountain ranges appeared in the main text are shown. In (A)–(D), a polygon represents a core area and the numbers from 1 to 13 are the ranks of relict species richness and RWR following the order from high to low among the core areas corresponding to Supplementary Tables 3–6. Abbreviations: D: Daba Mts; Dl: Daliang Mts; Dlo: Dalou Mts; Dy: Duyang Mts; H: Hoang Lien Son Mts; Him: Himalayas Mts; Lm: Longmen Mts; Lz: Liuzhao Mts; Ml: Miaoling Mts; Qln: Qinling Mts; W: Wu Mts; Wm: Wumeng Mts. For the national boundary between China and India, please see Supplementary Fig. 2A and Methods. Maps were generated using the software ArcGIS v. 10.5 (ESRI, Redlands, CA, USA) and modified using Canvas 12 (ACD Systems of America, Inc., Seattle, WA, USA). Map layers were obtained from site www.gadm.org.

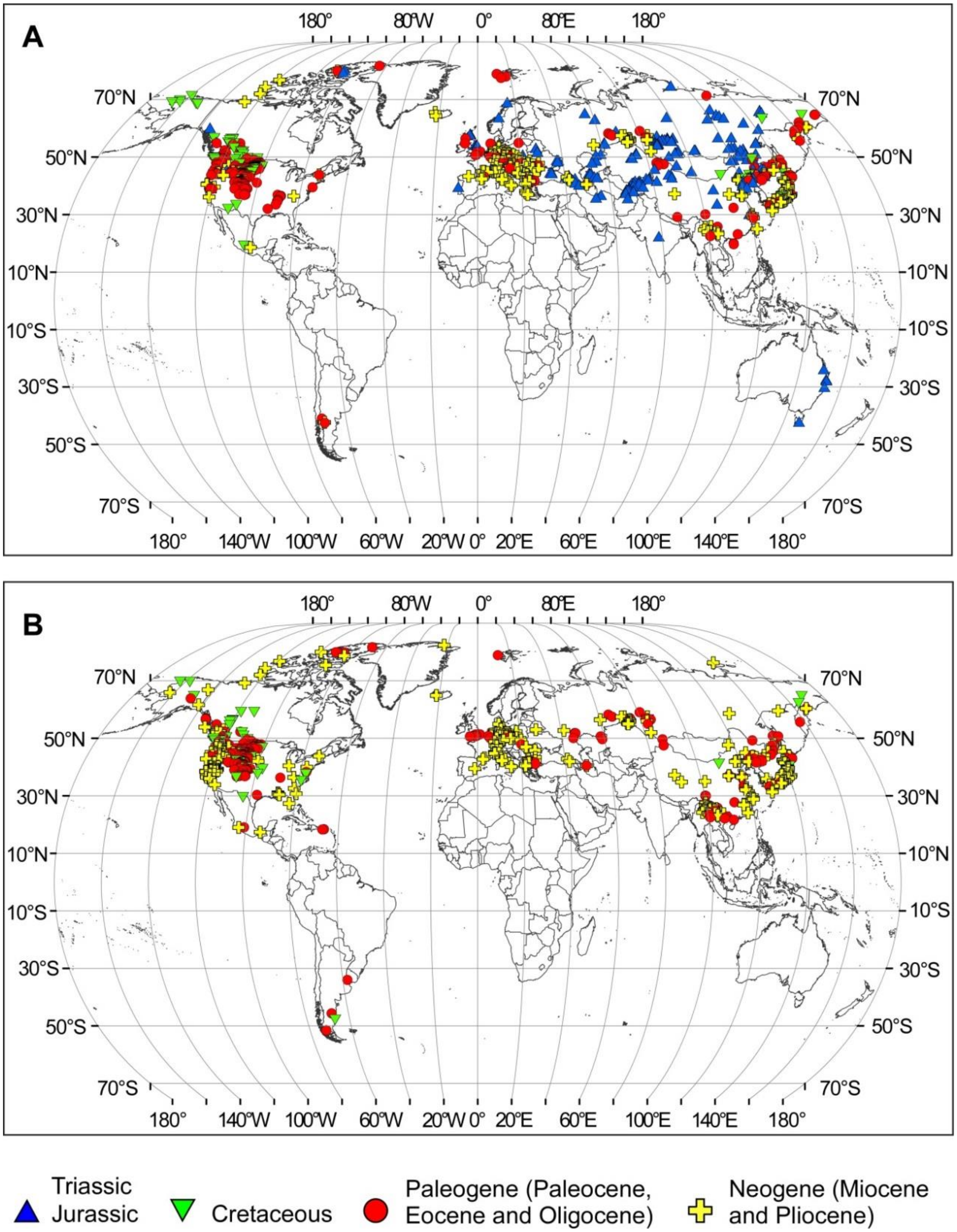

Supplementary Fig. 5 Historic (prior to the Pleistocene) distribution of the relict species in space and time. (A) Relict species of genera endemic to East Asia. (B) Relict species of genera having disjunct distributions between East Asia and other parts of the world. For the national boundary between China and India, please see Supplementary Fig. 2A and Methods. Maps were generated using the software ArcGIS v. 10.5 (ESRI, Redlands, CA, USA) and modified using Canvas 12 (ACD Systems of America, Inc., Seattle, WA, USA). Map layers were obtained from site www.gadm.org.

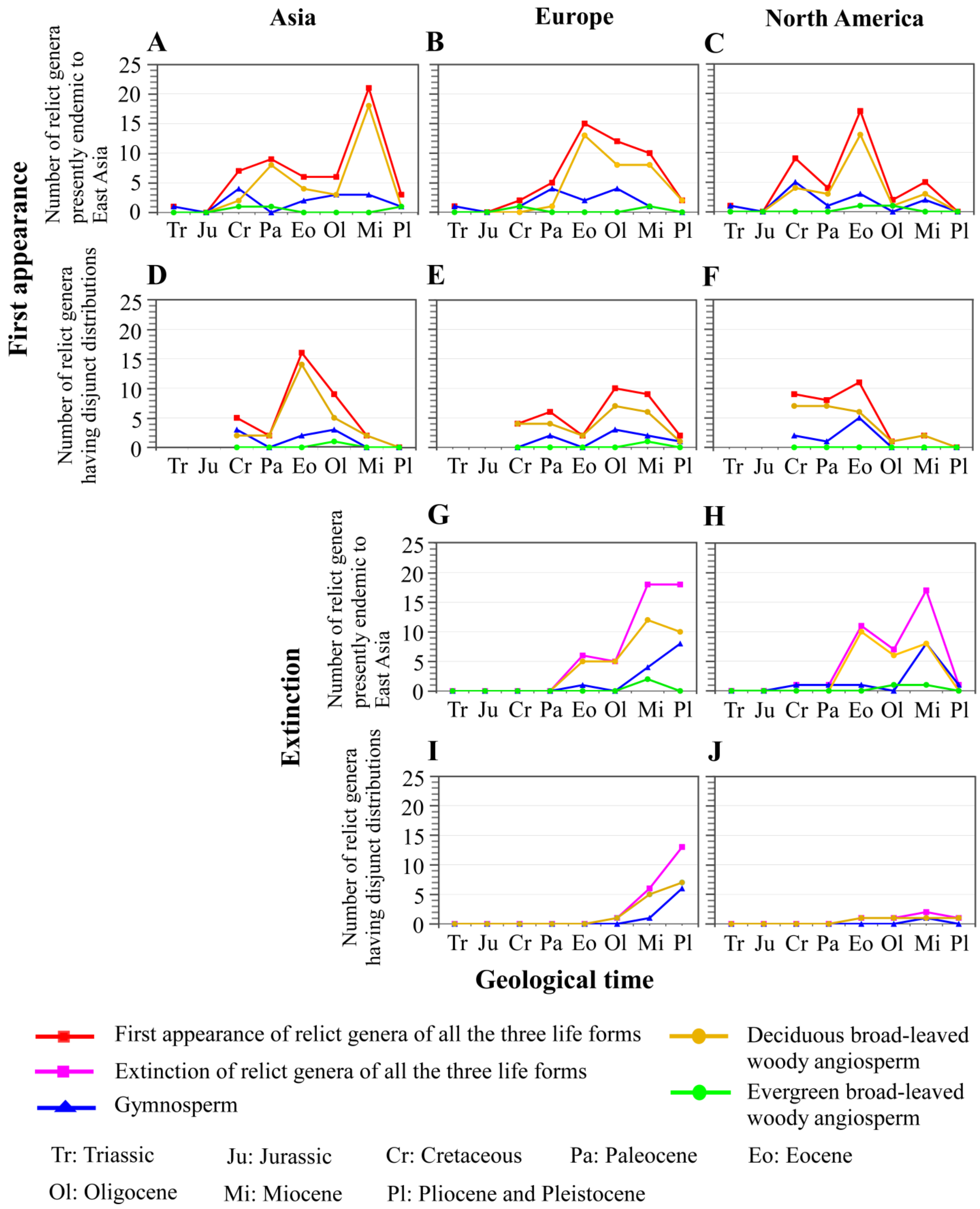

Supplementary Fig. 6 Changes in number of relict genera through geological time. (A)–(F) The number of the relict genera first appearing in Asia, Europe and North America. (G)–(J) The number of the relict genera that became extinct in Europe and North America.

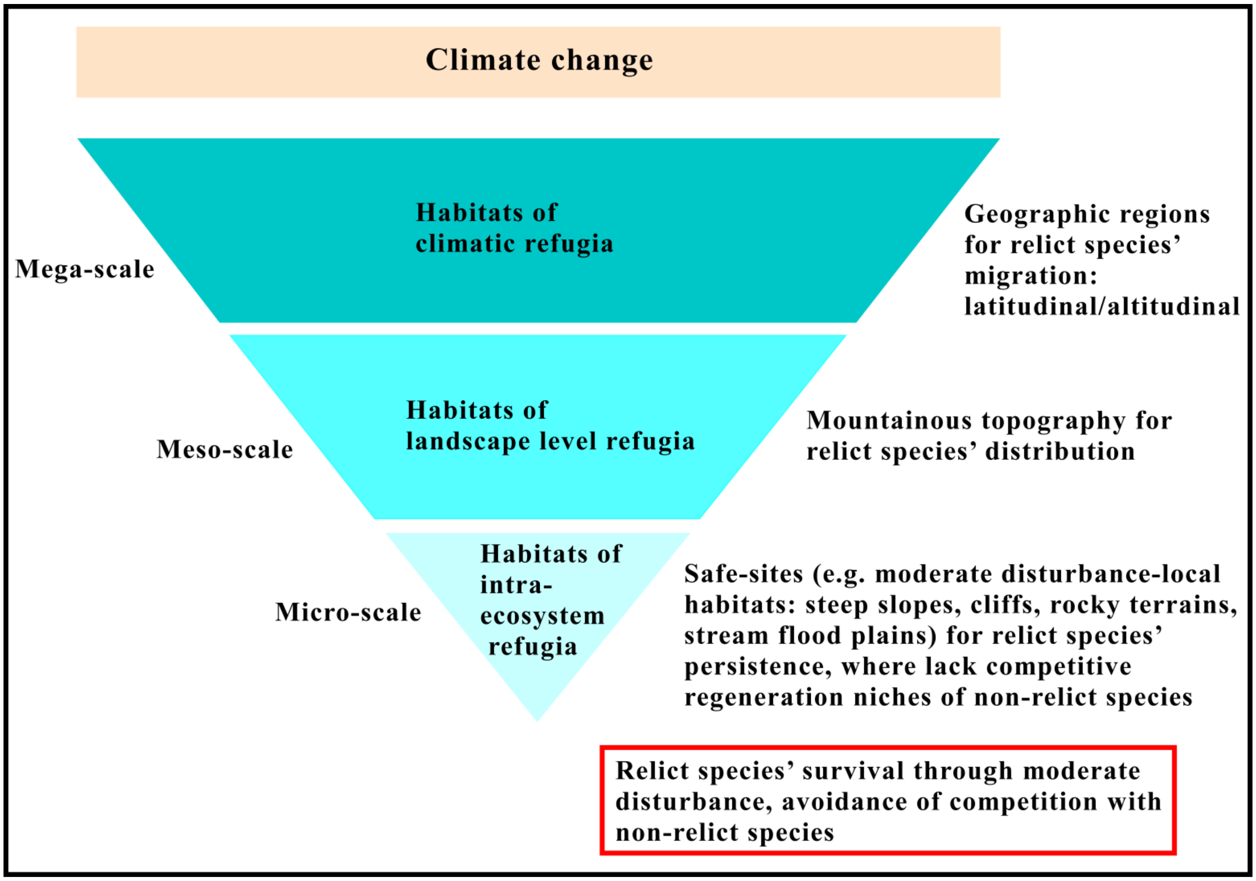

Supplementary Fig. 7 Schematic diagram of hierarchical habitat structure of refugia for relict plant species in East Asia.

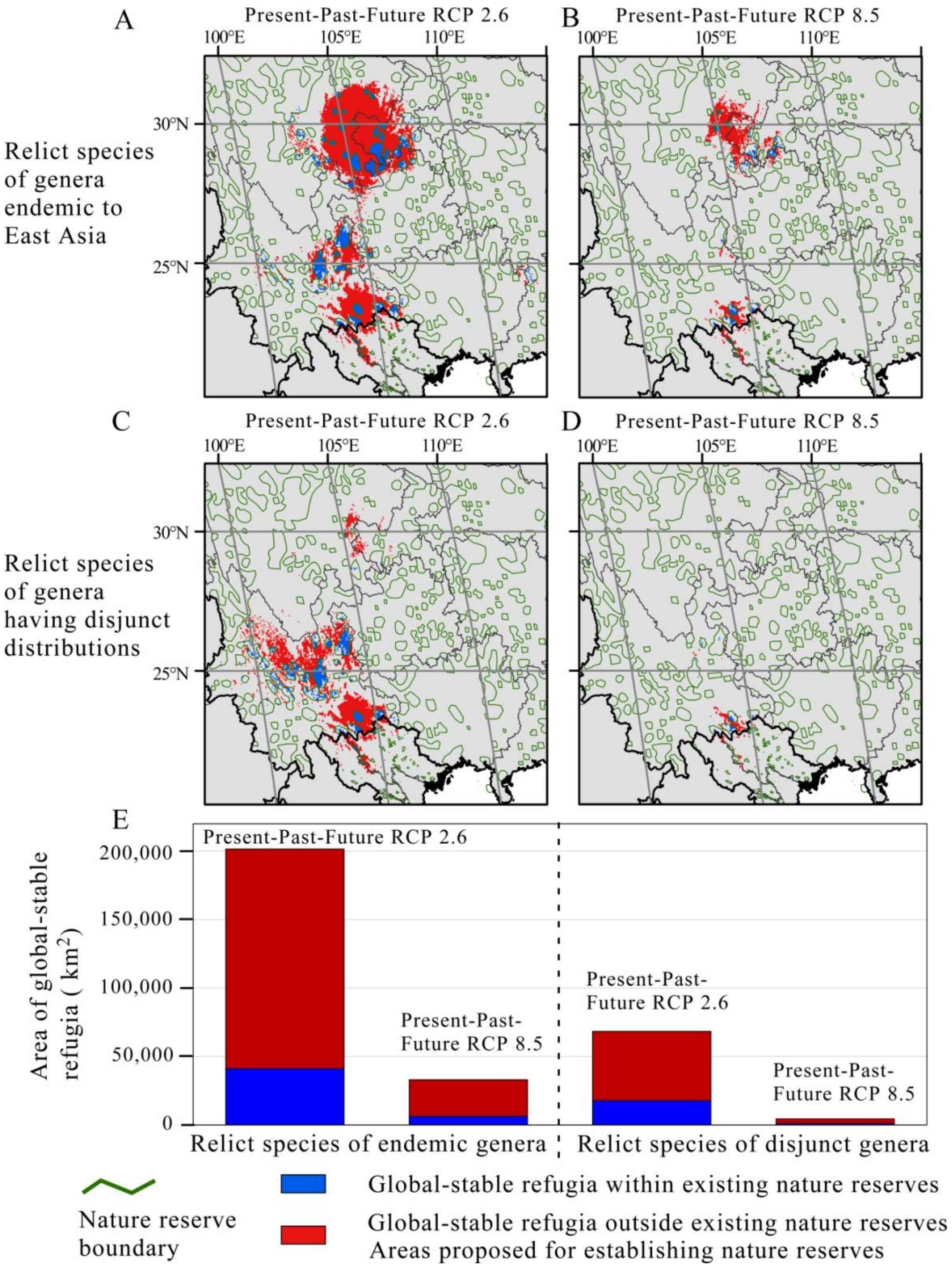

Supplementary Fig. 8 Modeled long-term stable refugia within and outside existing protected areas (nature reserves) under climatic scenarios, with areas proposed for conservation. (A)–(B) For relict species of genera endemic to East Asia: (A) under scenarios of present-past-future RCP 2.6, (B) under scenarios of present-past-future RCP 8.5. (C)–(D) For relict species of genera having disjunct distributions: (C) under scenarios of present-past-future RCP 2.6, (D) under scenarios of present-past-future RCP 8.5. (E) Areas of long-term stable refugia under various scenarios. Maps were generated using the software ArcGIS v. 10.5 (ESRI, Redlands, CA, USA) and modified using Canvas 12 (ACD Systems of America, Inc., Seattle, WA, USA). Map layers were obtained from site www.gadm.org.

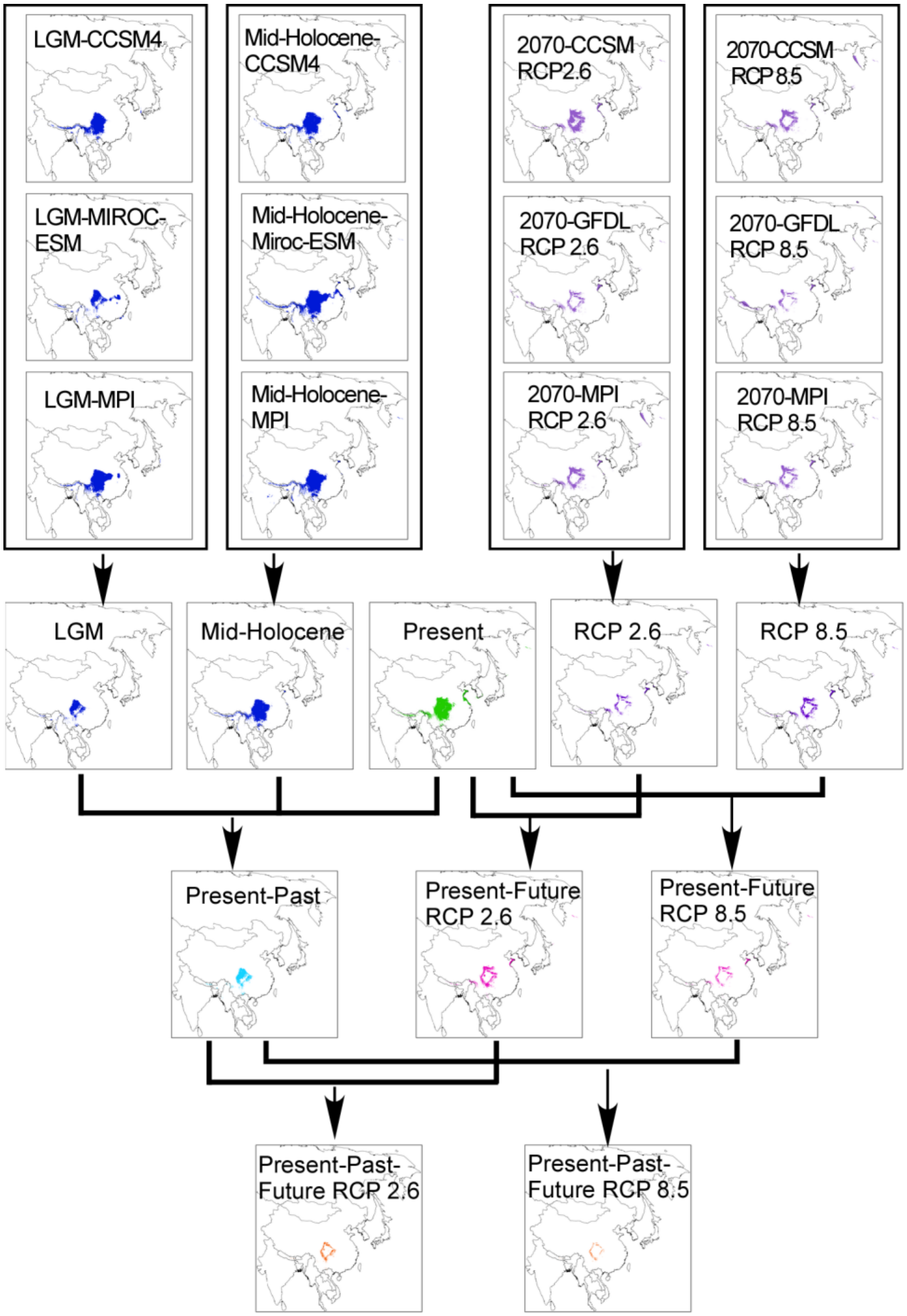

Supplementary Fig. 9 Assessment of five different types of time combinations (showing relict *Davidia involucrata* as an example). LGM: the overlap of the three LGM models; Mid-Holocene: the overlap of the three Mid-Holocene models; RCP 2.6: the overlap of the three 2070 RCP 2.6 models; RCP 8.5: the overlap of the three 2070 RCP 8.5 models; Present-Past: the overlap of the present with all the past models; Present-Future RCP 2.6: the overlap of the present with all the future models of RCP 2.6; Present-Future RCP 8.5: the overlap of the present with all the future models of RCP 8.5; Present-Past-Future RCP 2.6: the overlaps of the Present-Past with the Present-Future RCP 2.6; Present-Past-Future RCP 8.5: the overlap of the Present-Past with the Present-Future RCP 8.5. Maps were generated using the software ArcGIS v. 10.5 (ESRI, Redlands, CA, USA) and modified using Canvas 12 (ACD Systems of America, Inc., Seattle, WA, USA). Map layers were obtained from site www.gadm.org.

Supplementary Table 1 Relict genera endemic to East Asia.
Ev.=evergreen; Dec.=deciduous; Semi-Ev.=semi-evergreen; Sub-Ev.=sub-evergreen
Country code: AU=Australia, BG=Bulgaria, CA=Canada, CN=China, DE=Germany, FR=France, GE=Georgia, HU=Hungary, IE=Ireland, JP=Japan, KZ=Kazakhstan, MX=Mexico, PL=Poland, RU=Russia, UA=Ukraine, UK=United Kingdom, US=United States, UZ=Uzbekistan
~~~ indicates presence in none of the mega/macrofossil records, but having primitive morphologic characteristics or phylogenetic evidence that the genus is of Tertiary origin.
* Data sources: Please see the Methods section.

Extant genus	Life form	Date of earliest known fossil*	Country where the earliest fossil was found*
**Gymnosperm**			
Amentotaxus	Shrubs or small trees, Ev.	Cretaceous	CA
Cathaya	Trees, Ev.	Oligocene	DE
Cephalotaxus	Trees or shrubs, Ev.	Eocene	CN, DE
Cryptomeria	Trees, Ev.	Paleocene	IE
Cunninghamia	Trees, Ev.	Cretaceous	CN, JP,US, CA
Fokienia	Trees, Ev.	Miocene	CN
Ginkgo	Trees, Dec.	Triassic	CN, RU, AU, GE, UZ, UA, CA
Glyptostrobus	Trees, Dec.	Cretaceous	CN, JP, US, CA
Keteleeria	Trees, Ev.	Eocene	CA
Metasequoia	Trees, Dec.	Cretaceous	CN, JP, US, CA
Nothotsuga	Trees, Ev.	Miocene	DE
Pseudolarix	Trees, Dec.	Eocene	CN, JP, DE, CA, US
Pseudotaxus	Shrubs, Ev.	~~~	~~~
Sciadopitys	Trees, Ev.	Paleocene	CA
Taiwania	Trees, Ev.	Cretaceous	US, CA, RU
Thujopsis	Trees, Ev.	Miocene	JP
**Angiosperm**			
Akebia	Woody lianas, Dec. or Sub-Ev.	Miocene	JP, DE
Alcimandra	Trees, Ev.	~~~	~~~
Amesiodendron	Trees, Ev.	~~~	~~~
Ammopiptanthus	Shrubs, Ev.	~~~	~~~
Apterosperma	Shrubs or small trees, Ev.	~~~	~~~
Archiboehmeria	Shrubs or subshrubs, Dec.	~~~	~~~
Bretschneidera	Trees, Dec.	~~~	~~~
Burretiodendron	Trees, Dec. or Semi-Ev.	Miocene	CN
Camptotheca	Trees, Dec.	Oligocene	JP
Cercidiphyllum	Trees, Dec.	Cretaceous	US
Choerospondias	Trees, Dec.	Eocene	UK
Corylopsis	Shrubs or small trees, Dec.	Cretaceous	CN
Craigia	Trees, Dec.	Paleocene	RU
Cyclocarya	Trees, Dec.	Paleocene	US
Davidia	Trees, Dec.	Cretaceous	CA
~~~

Decaisnea	Shrubs, Dec.	Eocene	DE
Dipelta	Shrubs, Dec.	Eocene	US, UK
Dipentodon	Shrubs or trees, Semi-Ev.	~~~	~~~
Diplopanax	Trees, Ev.	Eocene	US, CA
Dipteronia	Trees, Dec.	Paleocene	US, RU
Disanthus	Shrubs, Dec.	Paleocene	JP, DE
Emmenopterys	Trees, Dec.	Eocene	US, DE
Engelhardia	Trees, Dec.	Eocene	US
Eucommia	Trees, Dec.	Eocene	CN, JP, KZ, US, CA
Euptelea	Trees or shrubs, Dec.	Eocene	US
Euryale	Perennial herbs, aquatic	Miocene	RU
Eurycorymbus	Trees, Dec.	~~~	~~~
Euryodendron	Trees, Ev.	~~~	~~~
Euscaphis	Small trees or shrubs, Dec.	Oligocene	DE
Exbucklandia	Trees, Ev.	Paleocene	CN
Fortunearia	Shrubs or small trees, Dec.	Oligocene	DE
Hemiptelea	Shrubs or trees, Dec.	Eocene	CN
Heptacodium	Shrubs or small trees, Dec.	~~~	~~~
Hovenia	Trees or shrubs, Dec.	Eocene	CA, US
Idesia	Trees, Dec.	Eocene	JP, KZ,
Kalopanax	Trees, Dec.	Eocene	RU
Kmeria	Trees, Ev.	~~~	~~~
Koelreuteria	Trees, Dec.	Cretaceous	CA
Kolkwitzia	Shrubs, Dec.	~~~	~~~
Loropetalum	Trees or shrubs, Dec.	Miocene	JP
Loxococcus	Palm trees, Ev.	~~~	~~~
Manglietiastrum	Trees, Ev.	~~~	~~~
Melliodendron	Trees or shrubs, Dec.	Miocene	JP
Monimopetalum	Scandent shrubs, Semi-Ev.	~~~	~~~
Ostryopsis	Shrubs, Dec.	~~~	~~~
Parakmeria	Trees, Ev.	~~~	~~~
Parapyrenaria	Trees, Ev.	~~~	~~~
Paulownia	Trees, Dec. or Ev.	Miocene	JP, DE
Phellodendron	Trees, Dec.	Oligocene	DE
Piptanthus	Shrubs, Ev.	~~~	~~~
Platycarya	Trees or shrubs, Dec.	Cretaceous	CN, MX, US
Platycladus	Trees or shrubs, Dec.	Miocene	CN
Poliothyrsis	Trees, Dec.	~~~	~~~
Potaninia	Shrublets, Dec.	~~~	~~~
Pteroceltis	Trees, Dec.	Eocene	US
Pterostyrax	Trees or shrubs, Dec.	Oligocene	DE
Rehderodendron	Trees, Dec.	Eocene	UK
Rhodoleia	Trees or shrubs, Ev.	Cretaceous	DE

Rhoiptelea	Trees, Dec.	~~~	~~~
Sargentodoxa	Shrubs, climbing, Dec.	Eocene	US, DE
Schizophragma	Shrubs or scandent shrubs, Dec.	Miocene	JP
Semiliquidambar	Trees, Dec. or Ev.	~~~	~~~
Sinofranchetia	Woody lianas, Dec.	~~~	~~~
Sinojackia	Trees or shrubs, Dec.	~~~	~~~
Sinomenium	Woody vines, Ev.	Miocene	JP, US, FR, GE
Sinopanax	Shrubs or small trees, Ev.	~~~	~~~
Sinowilsonia	Shrubs or small trees, Ev.	~~~	~~~
Skimmia	Shrubs or trees, Ev.	Miocene	BG
Tapiscia	Trees, Dec.	Eocene	US, DE
Tetracentron	Trees, Dec.	Paleocene	CN, JP, RU
Tetraena	Shrubs, Dec.	~~~	~~~
Tetrapanax	Shrubs or small trees, Ev.	~~~	~~~
Toricellia	Trees or shrubs, Dec.	Paleocene	US
Trapella	Herbs, aquatic	Miocene	JP, HU
Tripterygium	Scandent shrubs, Dec.	Pliocene	JP
Trochodendron	Trees or shrubs, Ev.	Cretaceous	JP
Weigela	Shrubs, Dec.	Oligocene	RU

Supplementary Table 2 Relict genera with disjunct distributions between East Asia and other parts of the world.

Ev.=evergreen; Dec.=deciduous; Semi-Ev.=semi-evergreen; Sub-Ev=sub-evergreen
Country code:
AR=Argentina, BE=Belgium, CA=Canada, CN=China, DE=Germany, JP=Japan, KZ=Kazakhstan, RU=Russia, UK=United Kingdom, US=United States
~~~ indicates presence in none of the mega/macrofossil records, but having primitive morphologic characteristics or phylogenetic evidence that the genus is of Tertiary origin.
* Data sources: Please see the Methods section.

Extant genus	Life form	Date of earliest known fossil*	Country where the earliest fossil was found*
**Gymnosperm**			
Calocedrus	Trees, Ev.	Eocene	CA
Cedrus	Trees, Ev.	Cretaceous	US, RU
Chamaecyparis	Trees, Ev.	Paleocene	RU, KZ
Pseudotsuga	Trees, Ev.	Eocene	US
Taxus	Trees or shrubs, Ev.	Cretaceous	CN
Thuja	Trees or shrubs, Ev.	Cretaceous	JP
Torreya	Trees or shrubs, Ev.	Cretaceous	US
Tsuga	Trees, Ev.	Eocene	US, CA
Xanthocyparis	Trees, Ev.	~~~	~~~
~~~

**Angiosperm**			
Aesculus	Trees or shrubs, Dec.	Paleocene	US, CA
Calycanthus	Shrubs, Dec.	Miocene	DE
Carya	Trees, Dec.	Cretaceous	US
Castanea	Trees or shrubs, Dec.	Paleocene	US, CA
Catalpa	Trees, Dec.	Eocene	CN, US
Corylus	Shrubs or trees, Dec.	Paleocene	CN, US
Fagus	Trees, Dec.	Cretaceous	DE, US
Gleditsia	Trees or shrubs, Dec.	Eocene	JP, US
Gymnocladus	Trees, Dec.	Miocene	CN, RU
Halesia	Trees or shrubs, Dec.	Eocene	US
Hamamelis	Shrubs or small trees, Dec.	Eocene	JP, US
Juglans	Trees or shrubs, Dec.	Cretaceous	US, CA
Liquidambar	Trees, Dec.	Cretaceous	US, AR
Liriodendron	Trees, Dec.	Cretaceous	JP, DE, US, CA
Nanophyton	Cushion subshrubs, Dec.	~~~	~~~
Nyssa	Trees, Dec.	Cretaceous	US
Ostrya	Trees, Dec.	Cretaceous	DE
Pachysandra	Subshrubs or perennial herbs, Ev.	~~~	~~~
Paliurus	Trees or shrubs, Ev. or Dec.	Paleocene	CA
Parrotia	Trees, Dec.	Eocene	JP
Parthenocissus	Woody lianas, Dec. or Semi-Ev.	Paleocene	BE, US
Periploca	Shrubs scandent, Dec. or Ev.	Eocene	BE
Platanus	Trees, Dec. or Semi-Ev.	Cretaceous	RU, US, CA
Pterocarya	Trees, Dec.	Cretaceous	DE
Reevesia	Trees, Dec. or Ev.	Oligocene	JP
Sambucus	Shrubs or perennial herbs, Ev. or Dec.	Paleocene	UK
Sassafras	Trees, Dec.	Cretaceous	US, CA, AR
Staphylea	Small trees or shrubs, Dec.	Miocene	JP, US
Trigonobalanus	Trees, Ev.	~~~	~~~
Wisteria	Woody lianas, Dec.	Oligocene	RU
Zelkova	Trees, Dec.	Eocene	CN, JP, US, CA

Supplementary Table 3 Core areas of relict species richness of genera endemic to East Asia. The latitudinal and longitudinal ranges of core areas correspond to the polygons in Supplementary Fig. 4A.

Rank	Geographic region where core area is situated	Species richness	Latitudes	Longitudes	Elevational range	Major mountain ranges
1	Southwestern China	144	23–35	101–110	480–2700	Liupan Mts, Qinling Mts, Daba Mts, Longmen Mts, Qionglai Mts, Daliang Mts, Wumeng Mts, Wulianfeng Mts, Wu Mts, Huaying Mts, Dalou Mts, Wulin Mts, Miaoling Mts, Jiuwandashan Mts, Fenghuang Mts, Duyang Mts, Liuzhao Mts, Jinzhong Mts
2	South-central China	108	26–34	110–113	380–2200	Xionger Mts of Qinling Mts, Funiu Mts of Qinling Mts, Daba Mts, Wu Mts, Wulin Mts, Xuefeng Mts
3	Southern China	102	22–26	110–116	550–1990	Nanling Mts, Dayao Mts, Yunkai Mts, Yunwu Mts, Jiulian Mts, Wuyi Mts, Luofu Mts
4	Northen Vietnam-Southwestern China	100	22–24	103–108	320–2650	Liuzhao Mts, Gongmu Mts, Daqing Mts, Hoang Lien Son Mts, Cao Bang Mountains/Hills
5	Southeastern China	88	25–33	116–122	270–1900	Tianmu Mts, Huaiyu Mts, Xianxialing Mts, Wuyi Mts, Yu Mts, Daimao Mts
6	South-central China	80	26–32	114–117	460–1700	Dabie Mts, Mufu Mts, Wugong Mts, Zhuguang Mts
7	Boundary of NW Yunnan, Tibet and Myanmar	62	25–29	98–100	1250–3100	Gaoligong Mts, Nu Mts, Yunling Mts
8	Southwestern China	44	24–26	100–101	1200–2800	Wuliang Mts, Ailao Mts
9	South-central to Central Japan	39	33–37	133–140	85–2220	Shikoku Mts, Chugoku Mts, Kii Mts, Hida Mts, Kiso Mts, Akaishi Mts, Echigo Mts,
10	Southern Japan	30	32–34	130–131	30–1510	Kyushu Mts

Supplementary Table 4 Core areas of rarity-weighted richness (RWR) of relict species of genera endemic to East Asia.

The latitudinal and longitudinal ranges of core areas correspond to the polygons in Supplementary Fig. 4B.

Rank	Geographic region where core area is situated	RWR	Latitudes	Longitudes	Elevational range	Major mountain ranges
1	Northern Vietnam-Southwestern China	7.89	19–24	103–108	450–2600	Liuzhao Mts, Gongmu Mts, Daqing Mts, Hoang Lien Son Mts, Tay Con Linh Mts, Con Voi Mts, Cao Bang Mountains/Hills
2	Southwestern China	6.45	24–32	105–110	450–2500	Daba Mts, Wu Mts, Dalou Mts, Wulin Mts, Miaoling Mts, Jiuwandashan Mts, Fenghuang Mts, Duyang Mts, Dayao Mts, Jinzhong Mts
3	Boundary of NW Yunnan and Myanmar	5.32	25–29	97–100	1200–3100	Yunling Mts, Nu Mts, Gaoligong Mts
4	Southwestern China	4.66	25–33	101–105	675–2800	Daba Mts, Longmen Mts, Qionglai Mts, Daliang Mts, Wulianfeng Mts, Wumeng Mts, Lunan Mts
5	Southern China	4.45	23–27	110–115	350–1700	Nanling Mts, Dayao Mts, Jiulian Mts
6	South-central China	3.58	26–28	110–113	510–1640	Xuefeng Mts
7	Southeastern China	3.10	27–30	115–118	350–1900	Mufu Mts, Huaiyu Mts, Wuyi Mts
8	Taiwan	2.34	22–26	120–122	200–2500	Taiwan Mts, Central Mountain Range
9	South-central China	2.30	29–32	110–111	400–2000	Daba Mts, Wu Mts
10	Southeastern China	1.94	28–31	119–122	180–1300	Tianmu Mts, Xianxialing Mts
11	Southern China (Hainan)	1.85	18–20	108–111	300–1580	Wuzhi Mts
12	Central Japan	1.75	34–37	135–140	65–2220	Kii Mts, Hida Mts, Kiso Mts, Akaishi Mts, Echigo Mts
13	South-central Japan	1.02	33–35	132–134	50–1840	Chugoku Mts, Shikoku Mts

Supplementary Table 5 Core areas of relict species richness of genera having disjunct distributions between East Asia and other parts of the world. The latitudinal and longitudinal ranges of core areas correspond to the polygons in Supplementary Fig. 4C.

Rank	Geographic region where core area is situated	Species richness	Latitudes	Longitudes	Elevational range	Major mountain ranges
1	Southwestern China	93	24–35	104–110	600–2800	Qinling Mts, Daba Mts, Huaying Mts, Wu Mts, Dalou Mts, Wulin Mts, Miaoling Mts, Jiuwandashan Mts, Jinzhong Mts
2	Southern China	71	24–28	110–115	500–1990	Xuefeng Mts, Nanling Mts, Zhuguang Mts, Jiulian Mts
3	South-central China	68	28–34	110–113	600–2050	Daba Mts, Wulin Mts, Wu Mts, Funiu Mts of Qinling Mts
4	Northen Vietnam-Southwestern China	66	22–24	103–107	700–2200	Liuzhao Mts, Ailao Mts, Gongmu Mts, Daqing Mts, Hoang Lien Son Mts, Cao Bang Mts/Hills
5	Southwestern China	65	28–34	102–105	710–2900	Qinling Mts, Daba Mts, Longmen Mts, Qionglai Mts, Daliang Mts
6	Southwestern China	63	24–28	99–103	1300–3000	Yunling Mts
7	Southeastern China	61	27–33	116–122	270–1800	Tianmu Mts, Huaiyu Mts, Yu Mts, Wuyi Mts, Xianxialing Mts
8	South-central China	59	26–32	114–117	480–1850	Dabie Mts, Mufu Mts, Wugong Mts, Zhuguang Mts
9	Boundary of NW Yunnan, Tibet and Myanmar	36	26–29	98–99	1200–3100	Gaoligong Mts, Nu Mts, Yunling Mts
10	South-Central Japan	29	34–35	135–137	60–1850	Kii Mts
11	Central Japan	28	35–38	137–140	85–2500	Kiso Mts, Akaishi Mts, Echigo Mts

Supplementary Table 6 Core areas of rarity-weighted richness (RWR) of relict species of genera having disjunct distributions between East Asia and other parts of the world. The latitudinal and longitudinal ranges of core areas correspond to the polygons in Supplementary Fig. 4D.

Rank	Geographic region where core area is situated	RWR	Latitudes	Longitudes	Elevational range	Major mountain ranges
1	Northern Vietnam-Southwestern China	11.50	21–24	102–108	700–2800	Ailao Mts, Liuzhao Mts, Gongmu Mts, Daqing Mts, Hoang Lien Son Mts, Cao Bang Mountains/Hills
2	South-central China	4.61	29–32	108–111	800–2100	Daba Mts, Wu Mts
3	Southwestern China	3.85	25–28	99–103	1000–3000	Daxue Mts, Gongwan Mts, Lunan Mts
4	Boundary of NE India and Bangladesh	3.69	24–26	90–92	88–1380	Garo Khasi Jaintia Hills
5	Boundary of NW Yunnan and Myanmar	2.44	25–28	98–99	1300–3000	Gaoligong Mts, Nu Mts
6	Southwestern China	2.28	24–26	107–109	600–1860	Miaoling Mts, Jiuwandashan Mts
7	Taiwan	2.24	22–26	120–122	600–2900	Taiwan Mts, Central Mountain Range
8	Northern Vietnam-Central Laos	2.20	18–21	104–106	200–2000	Annamite Mts
9	Southwestern China	2.00	24–25	104–106	700–2340	Jinzhong Mts
10	Southern China	1.82	24–26	112–115	650–1790	Nanling Mts
11	Southeastern China	1.41	30–31	118–121	325–1200	Tianmu Mts
12	Southern China (Hainan)	0.93	18–19	109–111	300–1580	Wuzhi Mts
13	South-central Japan	0.86	34–35	135–137	60–1850	Kii Mts

Supplementary Table 7 Predicted areas as suitable for relict species richness categories of genera endemic to East Asia.

Model	Total predicted area (km^2) for 1-128 relict species	Predicted area (km^2) for 1–5 relict species	Predicted area (km^2) for 6–10 relict species	Predicted area (km^2) for 11–20 relict species	Predicted area (km^2) for 21–50 relict species	Predicted area (km^2) for 51–128 relict species
Present	42,228,432	30,883,451	4,023,176	3,189,459	2,477,900	1,654,446
Mid-Holocene-CCSM	40,129,340	28,842,461	4,064,817	2,979,558	2,874,397	1,368,107
Mid-Holocene-MIROC	40,051,398	27,121,248	4,683,275	3,576,731	3,217,226	1,452,918
Mid-Holocene-MPI	40,569,191	28,711,971	4,374,264	3,113,698	2,800,756	1,568,502
Average Holocene	*40,249,976*	*28,225,227*	*4,374,119*	*3,223,329*	*2,964,126*	*1,463,176*
LGM-CCSM	32,616,064	19,564,336	4,819,769	4,774,507	2,562,049	895,403
LGM-MIROC	36,926,218	24,685,140	3,524,284	4,709,516	3,516,317	490,961
LGM-MPI	42,418,133	28,310,729	5,373,201	4,398,969	2,898,483	1,436,751
Average LGM	*37,320,138*	*24,186,735*	*4,572,418*	*4,627,664*	*2,992,283*	*941,038*
2070-CCSM-RCP 2.6	42,804,262	30,185,811	5,008,867	3,518,588	2,429,565	1,661,431
2070-CCSM-RCP 8.5	44,129,453	28,937,049	6,131,838	4,956,738	2,675,336	1,428,492
2070-GFDL-RCP 2.6	41,213,439	26,523,368	6,332,447	4,249,670	2,999,117	1,108,837
2070-GFDL-RCP 8.5	45,929,391	25,111,921	10,111,091	6,852,553	3,428,110	425,716
2070-MPI-RCP 2.6	43,262,316	30,797,093	5,000,224	3,495,655	2,561,216	1,408,128
2070-MPI-RCP 8.5	44,028,723	28,865,871	6,167,938	4,838,569	3,021,191	1,135,154
Average 2070	*43,561,264*	*28,403,519*	*6,458,734*	*4,651,962*	*2,852,423*	*1,194,626*

Supplementary Table 8 Predicted areas as suitable for relict species richness categories of genera having disjunct distributions between East Asia and other parts of the world.

Model	Total predicted area (km^2) for 1-128 relict species	Predicted area (km^2) for 1–5 relict species	Predicted area (km^2) for 6–10 relict species	Predicted area (km^2) for 11–20 relict species	Predicted area (km^2) for 21–50 relict species	Predicted area (km^2) for 51–128 relict species
Present	39,053,735	20,917,095	8,850,149	4,575,361	4,372,861	338,269
Mid-Holocene-CCSM	39,368,983	21,459,269	8,888,251	4,160,894	4,611,016	249,553
Mid-Holocene-MIROC	40,927,557	22,632,276	8,244,619	4,843,251	4,803,309	404,102
Mid-Holocene-MPI	40,239,145	21,900,797	9,104,744	4,291,171	4,590,872	351,561
Average Holocene	*40,178,562*	*21,997,447*	*8,745,871*	*4,431,772*	*4,668,399*	*335,072*
LGM-CCSM	50,996,842	32,559,317	7,680,284	6,601,852	3,867,950	287,439
LGM-MIROC	52,901,800	36,202,305	6,169,412	6,148,151	4,305,456	76,476
LGM-MPI	59,816,001	39,409,290	9,275,256	6,327,552	4,417,011	386,892
Average LGM	*54,571,548*	*36,056,971*	*7,708,317*	*6,359,185*	*4,196,806*	*250,269*
2070-CCSM-RCP 2.6	40,281,157	20,403,849	9,455,050	5,719,214	4,513,263	189,781
2070-CCSM-RCP 8.5	44,524,574	22,841,233	9,378,157	7,405,454	4,763,090	136,640
2070-GFDL-RCP 2.6	45,642,122	22,386,417	11,809,973	6,663,600	4,714,227	67,905
2070-GFDL-RCP 8.5	47,681,840	21,597,030	12,277,317	9,611,794	4,173,121	22,578
2070-MPI-RCP 2.6	40,283,596	20,676,154	9,322,918	5,643,561	4,449,212	191,751
2070-MPI-RCP 8.5	43,644,520	22,511,641	9,105,272	7,386,156	4,601,767	39,684
Average 2070	*43,676,302*	*21,736,054*	*10,224,781*	*7,071,630*	*4,535,780*	*108,057*

Supplementary Table 9 Predicted overlapping suitable-areas for relict species richness categories of genera endemic to East Asia.

Model	Total predicted area (km^2) for 1-85 relict species	Predicted area (km^2) for 1–3 relict species	Predicted area (km2) for 4–8 relict species	Predicted area (km^2) for 9–14 relict species	Predicted area (km^2) for 15–25 relict species	Predicted area (km^2) for 26–85 relict species
Present-Past	14,194,353	7,741,070	3,061,877	1,862,623	1,010,684	518,099
Present-Future RCP 2.6	33,286,395	20,136,890	6,309,587	2,724,372	1,863,053	2,252,493
Present-Future RCP 8.5	29,253,686	17,443,520	6,556,242	2,464,561	1,749,121	1,040,242
Present-Past-Future RCP 2.6	12,532,900	6,963,832	3,391,652	1,325,251	614,253	237,912
Present-Past-Future RCP 8.5	11,244,631	6,515,275	3,463,941	880,547	332,335	52,533

Supplementary Table 10 Predicted overlapping suitable-areas for relict species richness categories of genera having disjunct distributions between East Asia and other parts of the world.

Model	Total Predicted area (km^2) for 1-85 relict species	Predicted area (km^2) for 1–3 relict species	Predicted area (km^2) for 4–8 relict species	Predicted area (km^2) for 9–14 relict species	Predicted area (km^2) for 15–25 relict species	Predicted area (km^2) for 26–85 relict
Present-Past	27,433,445	18,149,628	5,490,658	2,041,459	1,309,448	442,252
Present-Future RCP 2.6	37,750,221	17,191,443	10,777,980	5,013,568	3,269,645	1,497,585
Present-Future RCP 8.5	35,863,169	16,354,822	11,594,503	4,626,518	2,690,419	596,907
Present-Past-Future RCP 2.6	25,579,135	17,027,627	5,734,448	1,743,255	958,919	114,886
Present-Past-Future RCP 8.5	23,083,330	15,385,465	5,776,515	1,344,725	569,164	7,461

Supplementary Table 11 The predicted areas of the gloabal stable-refugia for relict species richness (26-85) included within the network of Protected Areas (nature reserves) in China and northern Vietnam.

	Predicted area (km^2) for 26-85 relict species	Inside Protected Areas (km^2 and %)	Outside Protected Areas (km^2 and %)
Present-Past-Future RCP 2.6 for relict species of endemic genera	201,556	41,142 (20.41)	160,414 (79.59)
Present-Past-Future RCP 8.5 for relict species of endemic genera	33,041	6,407 (19.39)	26,634 (80.61)
Intercontinental Present-Past-Future RCP 2.6 for relict species of disjunct genera	68,325	17,800 (26.05)	50,525 (73.95)
Intercontinental Present-Past-Future RCP 8.5 for relict species of disjunct genera	4,478	1,243 (27.76)	3,235 (72.24)

Supplementary References

1. Manchester, S. R., Chen, Z-D., Lu, A-M., Uemura, K. Eastern Asian endemic seed plant genera and their paleogeographic history throughout the Northern Hemisphere. *J. Syst. Evol.* **47,** 1–42 (2009).
2. Qian, H. A comparison of generic endemism of vascular plants between East Asia and North America. *Int. J. Plant Sci.* **162,** 191–199 (2001).
3. Kubitzki, K., Krutzsch, W. Origins of East and South East Asian plant diversity. In *Floristic Characteristics and Diversity of East Asian Plants* (eds. Zhang, A. & Wu, S.) 56-70 (Beijing, China: China Higher Education Press, 1996).
4. An, Z., Kutzbach, J. E., Prell, W. L. & Porter, S. C. Evolution of Asian monsoons and phased uplift of the Himalaya–Tibetan plateau since Late Miocene times. *Nature*, **411,** 62–66 (2001).
5. Liu, X. *et al.* Continental drift and plateau uplift control origination and evolution of Asian and Australian monsoons. *Sci. Rep.* **7**, 40344; doi: 10.1038/srep40344 (2017).
6. Ramstein, G., Fluteau, F., Besse, J. & Joussaume, S. Effect of orogeny, plate motion and land-sea distribution on Eurasian climate change over the past 30 million years. *Nature* **386,** 788–795 (1997).
7. van der Hammen, T., Wijmstra, T. A. & Zagwijn, W. H. The floral record of the Late Cenozoic of Europe. In *Late Cenozoic Glacial Ages* (ed. Turekian, K. K.) 391-424 (Yale University Press, New Haven, 1971).
8. Martinetto, E. *et al.* Late persistence and deterministic extinction of "humid thermophilous plant taxa of East Asian affinity" (HUTEA) in southern Europe. *Palaeogeogr. Palaeoclimatol. Palaeoecol*. **467,** 211-231 (2017).
9. Chen, F., Wünnemann, B. & Yu, Z. Holocene climate variability in arid Asia: nature and mechanisms. *Quat. Int*. **194,** 1–5 (2009).
10. Ramankutty, N. & Foley, J. A. Estimating historical changes in global land cover: Croplands from 1700 to 1992. *Global Biogeochem. Cycles* **13,** 997–1027 (1999).
