# Supplementary material for: Identifying long-term stable refugia for relict plant species in East Asia
Source: Nat Commun. 2018 Oct 26;9:4488. doi: 10.1038/s41467-018-06837-3 (PMC6203703; doi:10.1038/s41467-018-06837-3)
Supplement: Supplementary file 3 — Description of Additional Supplementary Files [file 41467_2018_6837_MOESM3_ESM.pdf]

Description of Additional Supplementary Files

File Name: Supplementary Data 1
Description: Relict species of genera endemic to East Asia

File Name: Supplementary Data 2
Description: Relict species of genera with disjunct distributions between East Asia and other parts of the world.

File Name: Supplementary Data 3
Description: Forest types containing relict species in East Asia, at least one of them being a dominant

File Name: Supplementary Data 4
Description: Herbaria, websites and literature for relict species' current distribution information
