# Supplementary material for: Identifying long-term stable refugia for relict plant species in East Asia
Source: Nat Commun. 2018 Oct 26;9:4488. doi: 10.1038/s41467-018-06837-3 (PMC6203703; doi:10.1038/s41467-018-06837-3)
Supplement: Supplementary file 7 — Supplementary Data 4 [file 41467_2018_6837_MOESM7_ESM.docx]

| **Supplementary Data 4 Herbaria, websites and literature for relict species' current distribution information.** |
| --- |
| **Physical herbaria** |
| Herbarium, Institute of botany, Chinese Academy of Sciences) (Beijing, China) |
| Herbarium, Kunming Institute of Botany, the Chinese Academy of Sciences (Kunming, China) |
| Herbarium, Yunnan University (Kunming, China) |
| Herbarium, Chengdu Institute of Biology, Chinese Academy of Sciences (Chengdu, China) |
| Botanical Herbarium, Institute of Biology, Guizhou Academy of Sciences (Guiyang, China) |
| Herbarium, Institute of Botany, Guangxi Zhuangzu Anonimimous Region and Chinese Academy of Sciences (Nanning, China) |
| Herbarium, Hunan University of Science and Technology (Xiangtan, China) |
| Herbarium of South China Botanical Garden (Guangzhou, China) |
| Herbarium, Lushan Botanical Garden, Jiangxi Province and Chinese Academy of Sciences (Jiujiang, China) |
| Herbarium, Wuhan Botanical Garden, Chinese Academy of Sciences (Wuhan, China) |
| Herbarium, Institute of Botany, Jiangsu Province and Chinese Academy of Sciences (Nanjing, China) |
| Botanical Herbarium, School of Life Sciences, Nanjing University (Nanjing, China) |
| Botanical Herbarium, College of Life and Envrionment Science Hangzhou Normal University (Hangzhou, China) |
| Herbarium of University of Tokyo (Tokyo, Japan) |
| Herbarium of Natural History Museum and Institute (Chiba, Japan) |
| HN-Herbarium of Institute of Ecology and Biological Resources, Vietnam Academy of Science and Technology (Hanoi, Vietnam) |
| Herbarium of Institute of Tropical Biology (Ho Chi Minh City, Vietnam) |
| Herbarium of the Institute of Biology and Soil Science (Vladivostok, Russia) |
| Botanical Garden-Institute (Vladivostok, Russia) |
| **Websites** |
| http://www.cvh.org. (Retrieved November 8, 2015-August 25, 2016) |
| [http://gbif.org. (Retrieved November 8, 2015-August 25, 2016)](http://gbif.org./) |
| http://taibif.tw. (Retrieved March 8, 2016-August 1, 2016) |
| [http://biodiversity.bt/ (Retrieved June 1, 2016-September 1, 2016)](http://biodiversity.bt/%20(Retrieved%20June%201,%202016-September%201,%202016)) |
| [http://indiabiodiversity.org/ (Retrieved June 1, 2016-September 1, 2016)](http://indiabiodiversity.org/%20(Retrieved%20June%201,%202016-September%201,%202016)) |
| [http://botany.si.edu/myanmar/ (Retrieved June 1, 2016-September 1, 2016)](http://botany.si.edu/myanmar/%20(Retrieved%20June%201,%202016-September%201,%202016)) |
| [http://www.worldbotanical.com/ (Retrieved June 1, 2016-September 1, 2016)](http://www.worldbotanical.com/%20(Retrieved%20June%201,%202016-September%201,%202016)) |
| http://threatenedconifers.rbge.org.uk. (Retrieved June 1, 2016-September 1, 2016) |
| http;//flora.huh.harvard.edu/china/(Retrieved January 1, 2016-February 2, 2017) |
| [http://botany.si.edu/myanmar/ (Retrieved June 1, 2016-November 1, 2016)](http://botany.si.edu/myanmar/%20(Retrieved%20June%201,%202016-November%201,%202016)) |
| [http://www.floraofnepal.org/ (Retrieved September, 2017)](http://www.floraofnepal.org/%20(Retrieved%20September,%202017)) |
| **Publications (we retain the original Chinese language for publications in Chinese for convenience in checking.)** |
| Averyanov , L. V. *et al*. Preliminary observation of native *Glyptostrobus pensilis* (Taxodiaceae) stands in Vietnam. *Taiwania* **54** (3), 191–212 (2009) |
| Chung, K. F., van der Werff, H. & Peng, C. I. Observations on the Floral Morphology of Sassafras Randaiense (Lauraceae). Ann. Mo. Bot. Gard. **97**(1), 1-10 (2010). |
| Ge, X.-J. *et al*. Low genetic diversity and significant population structuring in the relict Amentotaxus argotaenia complex (Taxaceae) based on ISSR fingerprinting. *J. Plant Res.* **118**, 415–422 (2005). |
| Grierson, A. J. C. & Long, D. G. Flora of Bhutan: including a record of plants from Sikkim Vol. 1, part 3. (Royal Botanic Garden, Edinburgh, 1983). |
| Grierson, A. J. C. Flora of Bhutan: including a record of plants from Sikkim and Darjeeling Vol. 2, part 1. (Royal Botanic Garden, Edinburgh, 1991). |
| Guha Bakshi, D. N. Flora of Murshidabad District, West Bengal, India. (Scientific Publisher, Jodhpur, 1984). |
| He, L.-Y. et al. Forest structure and regeneration of the Tertiary relict Taiwania cryptomerioides in the Gaoligong Mountains, Yunnan, southwestern China. Phytocoenologia **45**, 135–156 (2015). |
| Horikawa Y. Atlas of the Japanese Flora II: an introduction to plant sociology of East Asia. (Gakken, Tokyo, 1976). |
| Horikawa Y. Atlas of the Japanese Flora: an introduction to plant sociology of East Asia. (Gakken, Tokyo, 1972). |
| Kurata, S. & Hamaya, T. Distribution maps of Japanese trees and shrubs, Reprinted from Illustrated Important Forest Trees of Japan, Vol. 1 (Second Edition). (Japan Forest Technical Association, Tokyo, 1971). |
| Kurata, S. & Hamaya, T. Distribution maps of Japanese trees and shrubs II, Reprinted from Illustrated Important Forest Trees of Japan, Vol. 2 (Second Edition). (Japan Forest Technical Association, Tokyo, 1974). |
| Kurata, S. & Hamaya, T. Distribution maps of Japanese trees and shrubs, Reprinted from Illustrated Important Forest Trees of Japan, Vol. 3. (Japan Forest Technical Association, Tokyo, 1971). |
| Kurata, S. & Hamaya, T. Distribution maps of Japanese trees and shrubs, Reprinted from Illustrated Important Forest Trees of Japan, Vol. 4. (Japan Forest Technical Association, Tokyo, 1973). |
| Lei, X. Y. The spatial distribution of Glyptostrobus pensilis in Provincial Forest Park in Xianfengshan, Zhouning, Fujian. *Forestry Exploration and Design* **1**, 53–57 (2017) |
| Lin, J. Y. *et al*. Community characteristics and conservation of Trigonobalanus verticillata (Fagaceae) on Yinggeling, Hainan Island. *Acta Ecol. Sin.* **27** (6): 2230-2238 (2007). |
| Loc, P. et al. Native conifers of Vietnam-a review. *Pak. J. Bot.* **49** (5), 2037–2068 (2017). |
| Luo, S., Huang, Y., Cao, X. & Liu, L. Reproduction and protection of critically endangered species of Glyptostrobus pensilis in Yiyang County. *Jiangxi Science* **34** (2), 187–189 (2016). |
| Phengklai, C. Flora of Thailand, Vol. 6, part 1, 1993. (Forest Herbarium, Royal Forest Department, Bangkok, 1993) |
| Qian S. H. *et al*. (2016) Effective conservation measures are needed for wild Cathaya argyrophylla populations in China: Insights from the population structure and regeneration characteristics. For. Ecol. Manag. **361**, 358-367 (2016) |
| Santisuk, T. & Larsen, K. Flora of Thailand, Vol.10, part 3. Publisher: Forest Herbarium, Royal Forest Department, Bankok, 2010). |
| Tang, C. Q. & Ohsawa, M. Zonal transition of evergreen, deciduous and coniferous forests along the altitudinal gradient on a humid subtropical mountain, Mt. Emei, Sichuan, China. *Plant Ecol*. **133**, 63–78 (1997). |
| Tang, C. Q. & Ohsawa, M. Tertairy relic deciduous forests on a subtropical mountain, Mt. Emei, Sichuan, China. *Folia Geobot.* **37,** 93–106 (2002). |
| Tang, C. Q. *et al.* Population persistence of a Tertiary relict tree *Tetracentron* *sinense* on the Ailao Mountains, Yunnan, China. *J. Plant Res.* **126,** 651–659 (2013). |
| Tang, C. Q. et al. Survival of a Tertiary relict species: Liriodendron chinense (Magnoliaceae) in southern China, with special reference to village fengshui forests. *Am. J. Bot.* **100** (10), 2112-2119 (2013). |
| Tang, C. Q. *et al*. Community structure and survival of Tertiary relict species *Thuja sutchuenensis* (Cupressaceae) in the subtropical Daba Mountains, southwestern China. *PLoS ONE* **10(4),** e0125307; doi:10.1371/journal.pone.0125307 (2015). |
| Thomas, P., Sengdala, K., Lamxay, V. & Khou, E. New records of conifers in Cambodia and Laos. *Edinburgh J. Bot.* **64** (1), 1–9 (2006). |
| Watson, M. F. et al. (eds.) Flora of Nepal, Vol. 3. (Royal Botanic Garden, Edinburgh, 2011). |
| Wu, Z.-Y. et al. The spatial distribution pattern of the population of relict plant Glyptostrobus pensilis in Pingnan. *Subtropical Agriculture Res*. **4** (1), 36–39 (2008). |
| Zheng, S.-Q. et al. The endangering causes and protection strategies for Glyptostrobus pensilis, an endemic relict plant in China. *Subtropical Agriculture Res.* **7** (4): 217–220 (2011). |
| 西藏植物名录编辑组. 西藏植物名录 (西藏自治区科学技术委员会印, 拉萨, 1980). |
| 曹善寿主编. 菜阳河自然保护区 (云南科学技术出版社, 昆明, 2003). |
| 曹善寿主编. 糯扎渡自然保护区 (云南科学技术出版社, 昆明, 2004). |
| 陈天虎主编. 恩施市维管束植物名录 (湖北科学技术出版社, 武汉, 2007). |
| 丁炳录,康健主编. 大兴安岭资源植物 (气象出版社, 北京, 1994). |
| 丁汉波等. 武夷山自然保护区科学考察报告集 (福建科学技术出版社, 福州, 1993). |
| 董旭,张胜邦,张更权主编. 青海祁连山自然保护区科学考察集 (中国林业出版社, 北京, 2007). |
| 傅坤俊,王作宾. 西北野生有用植物手册 4册 (陕西人民出版社, 西安, 1960). |
| 高宝莼主编. 四川省重点保护珍贵树木图志 (四川民族出版社, 成都, 1992). |
| 广西大瑶山自然资源综合考察队著. 广西大瑶山自然资源考察 (学林出版社, 上海, 1988). |
| 广西花坪林区综合考察队编. 广西花坪林区综合考察报告 (山东科学技术出版社, 济南, 1986). |
| 国家环境保护局,中国科学院植物研究所编. 中国珍稀濒危保护植物名录 1册 (科学出版社, 北京, 1987). |
| 国家林业局中南林业调查规划设计院,湖南六步溪自然保护区管理局. 湖南六步溪自然保护区综合科学考察 (2006). |
| 韩剑准. 海南尖峰岭自然保护区综合科学考察报告 (2001). |
| 何友均著. 三江源自然保护区森林植物多样性及其保护研究 (中国林业出版社, 北京, 2008). |
| 胡嘉琪,梁师文. 黄山植物 （复旦大学出版社, 上海, 1996). |
| 胡启明主编. 香港稀有及珍贵植物 (香港特别行政区政府渔农自然护理署, 2003). |
| 湖北赛武当自然保护区科考组. 湖北赛武当自然保护区科学考察报告 (2008). |
| 湖南省林业调查规划设计院. 湖南小溪自然保护区自然资源综合科学考察报告 (2000). |
| 湖南舜皇山自然保护区综合考察队. 湖南舜皇山自然保护区综合科学考察报告集 (国家林业局中南林业调查规划设计院, 长沙, 2006). |
| 黄金玲,蒋得斌. 广西猫儿山自然保护区综合科学考察 (湖南科学技术出版社, 长沙, 2002). |
| 黄威廉主编. 贵州珍稀濒危植物 (中国环境科学出版社, 北京, 1989). |
| 贾大柱主编. 安徽省天马自然保护区科学考察报告 (金寨县林业局编印, 金寨, 1993). |
| 江海声,黄文忠. 海南省南湾自然保护区及其周边生物多样性 (广东科学技术出版社, 广州, 1998). |
| 蒋志刚. 陕西青木川自然保护区的生物多样性 (清华大学出版社, 北京, 2005). |
| 蒋志刚. 江西桃红岭梅花鹿国家级自然保护区生物多样性研究 (清华大学出版社, 北京, 2009). |
| 金孝锋,翁东明主编. 清凉峰植物 (浙江大学出版社, 杭州, 2009). |
| 靳淑英. 中国高等植物模式标本汇编(补编) (中国林业出版社, 北京, 1999). |
| 靳淑英编. 中国高等植物模式标本汇编 (科学出版社, 北京, 1994). |
| 靳淑英编. 中国高等植物模式标本汇编(补编二) (科学出版社, 北京, 2007). |
| 九江市林业局. 九江市林业局野生植物标本馆馆藏标本志要 (2004). |
| 雷明德编. 陕西植被 (科学出版社, 北京, 1999). |
| 李保国,何鹏举主编. 陕西周至国家级自然保护区生物多样性 (陕西科学技术出版社, 西安, 2007). |
| 李采兰,杨济中,高东藩,陈家明,李朝斗. 贵州民间药物(第一辑) (贵州人民出版社, 贵阳, 1965). |
| 李操主编. 四川白羊自然保护区综合科学考察 (四川科学技术出版社, 成都, 2010). |
| 李恒,郭辉军,刀志灵主编. 高黎贡山植物 (科学出版社, 北京, 2000). |
| 李恒. 独龙江地区植物 (云南科学技术出版社, 昆明, 1993). |
| 李宏伟主编. 白马雪山国家级自然保护区 (云南民族出版社, 昆明, 2003). |
| 李金水主编. 黄山珍稀植物 (中国林业出版社, 北京, 2006). |
| 李书春,李秾,吴诗华,刘秀梅,吴泽民,何明勋,吴诚和. 安徽木本植物 (安徽科学技术出版社, 合肥, 1983). |
| 李应武,侯惠敏,苏炳勋,卓正大,张国梁,孙继周,林璋德,刘迺发,高兆宁,许成,张嘏,包天祥. 六盘山自然保护区科学考察 (宁夏人民出版社, 银川, 1988). |
| 李战刚,康克功,吴振海主编. 陕西平河梁省级自然保护区综合科学考察与生物多样性研究 (陕西科学技术出版社, 西安, 2008). |
| 李战刚,任毅,王学杰. 陕西长青国家级自然保护区综合科学考察报告 (陕西科学技术出版社, 西安, 2006). |
| 李振基,吴小平,陈小麟,刘长明. 江西九岭山自然保护区综合科学考察报告 (科学出版社, 北京, 2009). |
| 李振宇,邱小敏. 广西九万山植物资源考察报告 (中国林业出版社, 北京, 1993). |
| 李振宇主编. 龙栖山植物 (中国科学出版社, 北京, 1994). |
| 李忠平,刘增力,遇宝成. 吉林汪清东北红豆杉自然保护区生物多样性 (北京出版社, 北京, 2006). |
| 梁建平主编. 广西珍稀濒危树种 (广西科学技术出版社, 南宁, 2001). |
| 林媚珍,卓正大. 广东南昆山植物区系的基本特征. *华南师范大学学报*(自然科学版) **2**, 74-79 (1996). |
| 林鹏. 福建省南靖南亚热带雨林自然保护区科学考察报告 (厦门大学出版社, 厦门, 1999). |
| 林鹏. 福建梁野山自然保护区综合科学考察报告 (厦门大学出版社, 厦门, 2001). |
| 林鹏主编. 福建天宝岩自然保护区综合科学考察报告 (厦门大学出版社, 厦门, 2002). |
| 林业部调查规划设计院,广东大雾岭自然保护区. 广东大雾岭国家级自然保护区综合考察报告文集 (林业部调查规划设计院,广东大雾岭自然保护区, 1997). |
| 林英主编. 井冈山自然保护区考察研究 (新华出版社, 北京, 1990). |
| 刘恩德,彭华著. 永德大雪山种子植物区系和森林植被研究 (云南科学技术出版社, 昆明, 2010). |
| 刘厚培,朱景郊主编. 南岭山区自然资源开发利用 (科学出版社, 北京, 1992). |
| 刘少英,章小平,曾宗永主编. 九寨沟自然保护区生物多样性 (四川科学技术出版社, 成都, 2007). |
| 刘胜祥,瞿建平. 湖北星斗山自然保护区科学考察集 (湖北科学技术出版社, 武汉, 2003). |
| 刘胜祥,瞿建平主编. 湖北七姊妹山自然保护区科学考察与研究报告( 湖北科学技术出版社, 武汉, 2006). |
| 刘诗峰,张坚. 佛坪自然保护区生物多样性研究与保护 (陕西科学技术出版社, 西安, 2003). |
| 刘小明,郭英荣,刘仁林主编. 江西齐云山自然保护区综合科学考察集 (中国林业出版社, 北京, 2010). |
| 刘信中,方福生. 江西武夷山自然保护区科学考察集 (中国林业出版社, 北京, 2001). |
| 刘信中,傅清. 江西马头山自然保护区科学考察与稀有植物群落研究 (中国林业出版社, 北京, 2006). |
| 刘信中,王琅主编. 江西省庐山自然保护区生物多样性考察与研究 (科学出版社, 北京, 2010). |
| 刘信中,吴和平. 江西官山自然保护区科学考察与研究 (中国林业出版社, 北京, 2005). |
| 刘信中,肖忠优,马建华主编. 江西九连山自然保护区科学考察与森林生态系统研究 (中国林业出版社, 北京, 2002). |
| 刘毅,张绍云. 滇南地区药用植物 (云南科学技术出版社, 昆明, 2010). |
| 刘毅,郑进主编. 香格里拉民族药图鉴 (云南科学技术出版社, 昆明, 2008). |
| 刘振良,周学仁. 山西树木志 (中国林业出版社, 北京, 2001). |
| 刘正宇. 重庆金佛山生物资源名录 (西南师范大学出版社, 重庆, 2010). |
| 陆益新,黄广宾. 广西特有植物的研究. *广西植物* **9**(1), 37-58 (1989). |
| 陆益新,黄广宾. 广西特有植物的研究(续一). *广西植物* **9**(2), 119-186 (1989). |
| 倪志诚,程树志. 西藏南迦巴瓦峰地区维管束植物区系 (北京科学技术出版社, 北京, 1992). |
| 宁世江,李锋,何成新. 生物多样性关键地区－广西元宝山科学考察研究 (广西科学技术出版社, 南宁, 2009). |
| 潘金贵,韦直主编. 浙江省九龙山自然保护区自然资源研究 (中国林业出版社, 北京, 1996). |
| 庞雄飞,张金泉. 广东石门台自然保护区综合科学考察报告 (英德市人民政府, 2008). |
| 庞雄飞主编. 广东南岭国家级自然保护区生物多样性研究 (广东科学技术出版社, 广州, 2003). |
| 彭国栋总编. 南投县植物资源 [台湾省特有生物研究保育中心(舜程彩色印刷公司), 台中市, 1983]. |
| 彭国栋总编. 彰化县植物资源 (台湾省政府农林厅台湾省特有生物研究保育中心, 1997). |
| 彭华. 滇中南无量山种子植物 (云南科学技术出版社, 昆明, 1998). |
| 彭少麟,陈万成主编. 广东珍稀濒危植物 (科学出版社, 北京, 2003). |
| 彭少麟,廖文波,王英永,贾凤龙,凡强,沈如江,李贞,吴金火,陈晖. 中国三清山生物多样性综合科学考察 (科学出版社, 北京, 2008). |
| 祁承经,林亲众. 湖南树木志 (湖南科学技术出版社, 长沙, 2001). |
| 祁承经. 湖南植物名录 (湖南科学技术出版社, 长沙, 1987). |
| 任毅,温站强,李刚. 陕西米仓山自然保护区综合科学考察报告 (科学出版社, 北京, 2008). |
| 山东农学院植物学教研编组. 泰山植物名录 (山东农学院科学研究部, 济南, 1964). |
| 陕西龙池自然保护区综合科学考察队. 陕西龙池自然保护区综合科学考察报告 (2007). |
| 陕西省林业厅编. 太白山自然保护区综合考察论文集 (陕西师范大学出版社, 西安, 1989). |
| 上官铁梁,马子清,谢树莲著. 山西省珍稀濒危保护植物 (中国科学技术出版社, 北京, 1998). |
| 税玉民,陈文红. 中国喀斯特地区种子植物 1 (滇东南部)册 (科学出版社, 北京, 2006). |
| 税玉民. 文山县老君山维管植物. (文山县林业局云南省林业学校, 1996). |
| 税玉民主编. 滇东南红河地区种子植物 (云南科学技术出版社, 昆明, 2003). |
| 四川省林业厅. 四川白水河自然保护区综合考察报告 (四川省林业厅, 成都, 2001). |
| 宋朝枢,刘胜祥主编. 湖北后河自然保护区科学考察集 (中国林业出版社, 北京, 1999). |
| 宋立鑫主编. 西藏察隅慈巴沟国家级自然保护区自然资源综合科学考察报告 (西藏自治区林业勘察设计研究院, 拉萨, 2000). |
| 孙航,周浙昆. 雅鲁藏布江大峡弯河谷地区种子植物 (云南科学技术出版社, 昆明, 2000). |
| 孙治宇,刘少英. 四川长沙贡玛自然保护区综合科学考察报告 (四川省林业科学研究院, 成都, 2005). |
| 覃海宁,刘演主编. 广西植物名录 (科学出版社, 北京, 2010). |
| 谭伟福. 广西岑王老山自然保护区生物多样性保护研究 (中国环境科学出版社, 北京, 2005). |
| 谭伟福. 广西十万大山自然保护区生物多样性及其保护体系 (中国环境科学出版社, 北京, 2005). |
| 屠玉麟. 贵州特有植物初步研究(二). *贵州林业科技* **19**(4), 71-78 (1991). |
| 屠玉麟. 贵州特有植物初步研究(一). *贵州林业科技* **19**(3), 68-80 (1991). |
| 屠玉麟. 贵州特有植物初步研究(三). *贵州林业科技* **20**(2), 69-80 (1992). |
| 万煜. 广西龙虎山自然保护区维管植物名录 (龙虎山自然保护区管理站, 1986). |
| 汪松,解炎. 中国物种红色名录 1卷 (高等教育出版社, 北京, 2004). |
| 王德群. 安徽省特有植物的分类、分布和药用类群. *中国中药杂志* **24**(8), 451-454 (1999). |
| 王发国,陈坚,邢福武,曾庆文,陈红锋. 东莞珍稀植物 (华中科技大学出版社, 武汉, 2010). |
| 王国宏,冯自诚. 甘肃木本植物区系中特有现象分析. *甘肃农业大学学报* **31**(3), 282-287 (1996). |
| 王良民著. 山西太宽河自然保护区植物多样性研究 (中国农业科学技术出版社, 北京, 2010). |
| 王玛丽,邢连喜,张国昌主编. 陕西化龙山自然保护区综合科学考察报告 (西安地图出版社发行, 西安, 2004). |
| 王青锋,葛继稳. 湖北九宫山自然保护区生物多样性及其保护 (中国林业出版社, 北京, 2002). |
| 王诗云,赵子恩,彭铺松,蒋祖德. 华中珍稀濒危植物及其保存 1册 (科学出版社, 北京, 1995). |
| 吴德邻,邢福武,李泽贤,叶华谷,陈炳辉,钟义. 海南及广东沿海岛屿植物名录 (科学出版社, 北京, 1994). |
| 吴德鄰主编. 香港植物名录 (香港特别行政区政府渔农自然护理属, 2002). |
| 吴黄豪,纪伟涛. 江西鄱阳湖国家级自然保护区研究 (中国林业出版社, 北京, 2002). |
| 吴金清,金义兴,赵子恩. 三峡库区大老岭植物多样性与保护 (中国水利水电出版社, 北京, 2008). |
| 吴金清,赵子恩,金义兴著. 三峡库区珍稀濒危保护植物彩色图谱 (中国水利水电出版社, 北京, 2009). |
| 西藏林业勘察设计研究所. 中国西藏羌塘国家级自然保护区总体规划 (1999). |
| 谢双喜,李明晶,喻理飞. 贵州朱家山自然保护区科学考察集 (中国林业出版社, 北京, 2000). |
| 谢双喜,喻理飞,周庆主编. 大沙河自然保护区本底资源 (贵州科学技术出版社, 贵阳, 2006). |
| 邢福武,吴德邻. 海南岛特有植物的研究. 热带亚热带植物学报 **3**(1), 1-12 (1995). |
| 邢福武. 中国的珍稀植物 (湖南教育出版社, 长沙, 2005). |
| 徐荣章. 天目山木本植物图鉴 (中国林业出版社, 北京, 1989). |
| 徐永椿,姜汉桥,全复主编. 西双版纳自然保护区综合考察报告集 (云南科学技术出版社, 昆明, 1987). |
| 徐志辉,李增耀. 中国云南红河州野生珍稀动植物 (云南科学技术出版社, 昆明, 2004). |
| 徐志辉主编. 怒江自然保护区 (云南美术出版社, 昆明 (1998). |
| 许建初. 云南绿春黄连山自然保护区 (云南科学技术出版社, 昆明, 2003). |
| 许天全,吴金清. 三峡库区地方特有维管植物研究. *武汉植物学研究* **18**(3), 253-256 (2000). |
| 薛纪如主编. 高黎贡山国家自然保护区 (中国林业出版社, 北京, 1995). |
| 闫丽春主编. 云南哀牢山种子植物 (云南科学技术出版社, 昆明, 2009). |
| 杨宇明,杜凡. 中国南滚河国家级自然保护区 (云南科学技术出版社, 昆明, 2004). |
| 杨宇明,杜凡. 云南铜壁关自然保护区科学考察研究 (云南科学技术出版社, 昆明, 2006). |
| 杨宇明,田昆,和世钧主编. 中国文山国家级自然保护区科学考察研究 (科学出版社, 北京, 2008). |
| 叶永忠,瞿文元,黄远超主编. 连康山自然保护区科学考察集 (科学出版社, 北京, 2002) |
| 叶永忠,汪万森,李合中. 河南小秦岭自然保护区科学考察集 (科学出版社, 北京, 2004). |
| 喻理飞,李明晶,谢双喜. 贵州佛顶山自然保护区科学考察集 (中国林业出版社, 北京, 2000). |
| 喻理飞,谢双喜,吴太伦. 宽阔水自然保护区综合科学考察集 (贵州科学技术出版社, 贵阳, 2004). |
| 喻庆国,钱德仁主编. 小黑山自然保护区 (云南科学技术出版社, 昆明, 2006). |
| 喻勋林,肖育檀. 湖南石灰岩特有植物的初步研究. *中南林学院学报* **19**(2), 34-38 (1999). |
| 臧得奎,樊金会. 山东省特有植物的研究. *植物研究* **14**(1), 48-58 (1994). |
| 张光富. 安徽板桥自然保护区植物多样性 (南京师范大学出版社, 南京, 2007). |
| 张国珍,杨道德主编. 湖南壶瓶山国家级自然保护区科学考察报告集 (湖南科学技术出版社, 长沙, 2004). |
| 张海浪,刘国云,袁正科主编. 湖南省通道县龙底沟谷森林生态系统自然保护区自然资源研究 (湖南科学技术出版社, 长沙, 2003). |
| 张华海,李明晶,邓锦光. 黎平太平山自然保护区综合科学考察集 (贵州科学技术出版社, 贵阳, 2006). |
| 张华海主编. 贵州野生珍贵植物资源 (中国林业出版社, 北京, 2000). |
| 张华海主编. 老蛇冲自然保护区科学考察集 (贵州科学技术出版社, 贵阳, 2003). |
| 张华海主编. 南宫自然保护区科学考察集 (贵州科学技术出版社, 贵阳, 2003). |
| 张建军. 甘肃小陇山国家级自然保护区生物多样性 (北京出版社, 北京, 2009). |
| 张书东,王红,李德铢著. 滇东北巧家药山种子植物名录 (云南科学技术出版社, 昆明, 2008). |
| 张书理,韩力峰,王国文主编. 大黑山自然保护区综合科学考察集 (大黑山自然保护区管理局,敖汉旗环境保护局, 2000). |
| 张晓台,滕崇德编著. 山西野生植物 (山西人民出版社, 太原, 1960). |
| 张耀甲,彭泽祥. 白龙江流域珍稀、特有植物的多样性及其保护. *甘肃科学学报* **10**(2), 15-19 (1998). |
| 张炤玒,郎益贵,董福辰. 沂山植物 (山东大学出版社, 济南, 1992). |
| 赵之一. 内蒙古珍稀濒危植物图谱 (中国农业科学技术出版社, 北京, 1992). |
| 浙江省开化县林业局,古田山自然保护区. 浙江省古田山自然保护区动植物名录 (浙江省开化县林业局,古田山自然保护区管理处, 1999). |
| 浙江省科学技术协会编. 九龙山自然资源综合科学考察报告 (浙江省科学技术协会, 杭州, 1982). |
| 浙江省磐安县环境保护局. 浙江大盘山自然保护区自然资源综合考察报告 (浙江省磐安县环境保护局, 2001). |
| 郑成洋. 福建武夷山自然保护区珍稀、濒危和特有植物及其分布. *福建林业科技* **30**(3), 54-58 (2003). |
| 郑重,许天全. 湖北省珍稀特有植物及其分布概况. *环境科学与技术* **4**, 40-47 (1990). |
| 郑重,赵子恩,付书遐,刘启宏,郑洁华,黄仁煌,王映明,倪学明,张树藩,高文本,汪前生,王诗云,龙颜贞,王宁珠,姜钟华. 神农架植物 (湖北人民出版社, 武汉, 1980). |
| 重庆大风堡自然保护区科考组. 重庆大风堡自然保护区科学考察报告 (2005). |
| 周伟,陈宝昆. 云南碧塔海自然保护区 (云南科学技术出版社, 昆明, 2010). |
| 周政贤,姚茂森. 雷公山自然保护区科学考察集 (贵州人民出版社, 贵阳, 1989). |
| 朱华. 西双版纳石灰岩森林及植物多样性研究 (中国科学院西双版纳热带植物园, 1996). |
| 朱华. 西双版纳龙脑香热带雨林生态学与生物地理学研究 (云南科学技术出版社, 昆明, 2000). |
| 朱兆泉,宋朝枢. 神农架自然保护区科学考察集 (中国林业出版社, 北京, 1999). |
| 邹天才. 贵州特有及稀有种子植物 (贵州科学技术出版社, 贵阳, 2001). |
